# The PROM1^+^SMAD5^+^ Tumor-Initiating Subpopulation Shapes Premetastatic Niches through Spatial Multi-Omics Landscapes in HER2-Positive Breast Cancer

**DOI:** 10.34133/cancomm.0046

**Published:** 2026-09-09

**Authors:** Huijing Yin, Wei Wang, Jing Ge, Guangjian Fan, Jichao Shi, Ye Tian, Deping Kong, Fanyi Zhang, Jing Wei, Hongrui Zhan, Zhenyu Xu, Jintong Ge, Shuwei Wang, Zhiming Zhang, Yunna Zheng, Zhifa Zhu, Qingzhen Zi, Xiaohua Yao, Yanting Ding, Bo Song, Ruizhi Li, Liming Fan, Hao Yin, Yinghao Jiang, Shaoqian Zhang, Xingning Fu, Jing Wang, Yan Zhou, Haihua Xue, Yannan Qiu, Ling Ge, Yuqin Yang, Yinhui Jin, Binbin Lou, Shuning Ding, Tao Zhang, Denghui Wei, Li Zhu

**Affiliations:** ^1^ Precision Research Center for Refractory Diseases, Institute for Clinical Research, Shanghai General Hospital, Shanghai Jiao Tong University School of Medicine, Shanghai, P. R. China.; ^2^ Department of General Surgery, Shanghai General Hospital, Shanghai Jiao Tong University School of Medicine, Shanghai, P. R. China.; ^3^ Shanghai Immune Therapy Institute, Renji Hospital, Shanghai Jiao Tong University School of Medicine, Shanghai, P. R. China.; ^4^ School of Materials Science and Engineering, Shanghai Institute of Technology, Shanghai, P. R. China.; ^5^ Core Facility of Basic Medical Sciences, Shanghai Jiao Tong University School of Medicine, Shanghai, P. R. China.; ^6^ Becton Dickinson Medical Devices (Shanghai) Co., Ltd., Shanghai, P. R. China.; ^7^ OE Biotech Co., Ltd., Shanghai, P. R. China.; ^8^ Shanghai Maihui Biotechnology Co., Ltd., Shanghai, P. R. China.; ^9^ Akoya Biosciences, Marlborough, MA, USA.; ^10^ Department of Pathology, Weifang People’s Hospital, Weifang, Shandong, P. R. China.; ^11^ Pathology Center, Shanghai General Hospital, Shanghai Jiao Tong University School of Medicine, Shanghai, P. R. China.; ^12^ Central Laboratory, Renji Hospital, School of Medicine, Shanghai Jiao Tong University, Shanghai, P. R. China.; ^13^ Accuramed Biotechnology Co., Limited, Shanghai, P. R. China.; ^14^ Center for Excellence in Molecular Cell Science, Chinese Academy of Sciences, Shanghai, P. R. China.; ^15^ Department of Laboratory Animal Center, Shanghai General Hospital, Shanghai Jiao Tong University School of Medicine, Shanghai, P. R. China.; ^16^ Stomatology Department, Shanghai General Hospital, Shanghai Jiao Tong University School of Medicine, Shanghai, P. R. China.; ^17^ Department of Orthopedic Oncology, Shanghai General Hospital, Shanghai Jiao Tong University School of Medicine, Shanghai, P. R. China.; ^18^ Shanghai Bone Tumor Institution, Shanghai, P. R. China.; ^19^ State Key Laboratory of Oncology in South China, Guangdong Key Laboratory of Nasopharyngeal Carcinoma Diagnosis and Therapy, Guangdong Provincial Clinical Research Center for Cancer, Sun Yat-sen University Cancer Center, Guangzhou, Guangdong, P. R. China.

## Abstract

**Background:** Human epidermal growth factor receptor 2 (HER2)-positive breast cancer exhibits high metastatic potential, linked not only to intrinsic cancer cell traits but also to critical crosstalk with the tumor microenvironment. However, the coevolutionary mechanisms between cancer cells and multiple stromal subpopulations in driving distant metastasis remain poorly understood. Therefore, this study aimed to explore the microenvironmental regulatory mechanisms of breast tumor-initiating cells and their roles in HER2-positive breast cancer metastasis. **Methods:** Integrated multi-omics analyses (spatial transcriptomics, metabolomics, spatial in situ analysis, and proteomics) were used to identify novel cell subpopulations and their interactions. High-throughput sequencing of exosomal microRNAs (miRNAs) and single-nucleus RNA from the same tissue was performed to explore the molecular mechanisms underlying cell crosstalk. In vitro experiments were conducted to verify the interaction between stromal cells and prominin 1 (PROM1)^+^ SMAD family member 5 (SMAD5)^+^ cells. In vivo murine breast cancer models were established to confirm the role of stromal subpopulations in pulmonary metastasis, and parabiosis assays were carried out to compare key cell subpopulations between tumor-bearing mice and normal mice. Clinical samples were analyzed to correlate key cell subpopulations with clinicopathological features and prognosis. **Results:** A breast tumor-initiating subpopulation, PROM1^+^ SMAD5^+^ cells, and its interactions with stromal cells, specifically adiponectin (ADIPOQ)^+^ notch receptor 4 (NOTCH4)^+^ adipocytes and decorin (DCN)^+^ transmembrane 4 L six family member 1 (TM4SF1)^+^ fibroblasts, were identified by integrated multi-omics analyses. Mechanistically, these 2 stromal subpopulations delivered functional miRNAs and mediated coatomer protein complex subunit alpha (COPA)-dependent epidermal growth factor receptor (EGFR) activation in PROM1^+^SMAD5^+^ cells, thereby triggering the EGFR–SMAD5–cytochrome P450 family 3 subfamily A member 4 (CYP3A4) axis to induce partial epithelial–mesenchymal transition (pEMT) and metastasis. Additionally, stroma-secreted exosomal miR-671-3p down-regulated Claudin1 in PROM1^+^SMAD5^+^ cells, promoting their evolution into PROM1^+^SMAD5^+^Claudin1^−^ subpopulations with enhanced stemness and metastatic potential. In vivo experiments confirmed that the 2 stromal subpopulations markedly promoted pulmonary metastasis, and the 3 identified subpopulations preferentially accumulated in the primary tumors, lymph nodes, and pulmonary metastatic lesions of tumor-bearing mice. Clinically, these 3 subpopulations form a “trinity niche”, whose aggregation associated with HER2 positivity, high malignancy, and lymph node/pulmonary metastasis, and predicted poor prognosis. **Conclusion:** This study clarified the microenvironmental regulation of breast tumor-initiating cells and provided new insights into precision therapy.

## Background

Breast cancer surpassed lung cancer as the most commonly diagnosed malignancy worldwide in women since 2020, with 2.26 million new cases reported in 1 year [1]. Among its subtypes, human epidermal growth factor receptor 2 (HER2)-positive breast cancer is distinguished by its aggressive behavior, including high metastatic potential and poor prognosis [2]. Despite advances in anti-HER2 therapies, distant metastasis, particularly to the lung and lymph nodes, remains a major cause of treatment failure, with median survival of affected patients remaining under 30 months [3]. These observations underscore an urgent need to elucidate the mechanisms driving metastasis in HER2-positive breast cancer, in which both cancer cell-intrinsic properties and tumor microenvironment (TME) crosstalk are pivotal.

Metastasis is a complex, multistep process governed by dynamic interactions between cancer cells and their surrounding microenvironments. The “seed and soil” hypothesis [4] posits that disseminated cancer cells (“seeds”) require a permissive TME (“soil”) to colonize distant organs, whereas James Ewing’s blood flow hypothesis [5] emphasizes the influence of circulatory patterns on metastatic organotropism. In breast cancer, pulmonary metastasis is particularly prevalent, accounting for approximately 60% to 70% of disease-related deaths [6]; however, the specific “soil” factors that enable metastatic colonization remain poorly defined. Notably, accumulating evidence indicates that cancer cells may disseminate before the primary tumor becomes clinically detectable and enter a dormant state due to failure to transition from the “priming” to the “licensing” phase [7], highlighting the critical role of the premetastatic niche in metastatic success.

Tumor-initiating cells (TICs), or cancer stem cells (CSCs), are central drivers of metastasis owing to their stemness, plasticity, and adaptability [8]. In breast cancer, CSCs are classically identified by markers such as CD44^+^CD24^−^ [9] and aldehyde dehydrogenase 1 family member A3 (ALDH1A3)^+^ [10]; however, these subpopulations represent opposite extremes of the epithelial–mesenchymal transition (EMT) spectrum. Specifically, CD44^+^CD24^−^ cells tend to exhibit mesenchymal characteristics, whereas ALDH1A3^+^ cells predominantly retain epithelial traits [9]. This dichotomy constrains our understanding of TICs that may exist in hybrid epithelial–mesenchymal (E/M) states [11], which are increasingly recognized as critical for metastatic competence. Recent studies have identified a cytokeratin 14 (K14)^+^E-cadherin^+^ subpopulation capable of transitioning between epithelial and mesenchymal states, and functioning as “leader cells” in collective invasion and metastasis [12]. However, whether analogous pEMT-prone TICs exist in HER2-positive breast cancer and how they interact with stromal components to promote metastasis remain unresolved.

The TME, particularly stromal cell populations such as adipocytes and fibroblasts, is a key regulator of TIC behavior [13]. Adipocytes within breast tissue secrete cytokines and lipids that promote cancer cell proliferation and invasion [14], whereas cancer-associated fibroblasts remodel the extracellular matrix and secrete growth factors to facilitate dissemination [15,16]. However, most prior studies have focused on individual stromal cell types, largely overlooking the coordinated effects of multiple stromal subsets. In HER2-positive breast cancer, the coordinated roles of adipocytes and fibroblasts in shaping TIC stemness, EMT plasticity, and metastatic potential, through direct contact or paracrine mechanisms such as exosomal signaling, remain largely uncharacterized.

Exosomes have emerged as critical mediators of intercellular communication within the TME by transferring microRNAs (miRNAs) and proteins that reprogram recipient cells [17]. Both preclinical and clinical studies suggest that exosomes contribute to breast cancer progression and drug resistance [18]. Nevertheless, the role of exosomes in coordinating TIC–stromal interactions in HER2-positive breast cancer remains insufficiently explored. In parallel, advances in spatial transcriptomics and multi-omics technologies now enable in situ interrogation of cell–cell interactions, providing unprecedented resolution to map TME landscapes and identify novel cellular subpopulations.

Against this backdrop, the present study aimed to identify novel TIC subpopulations in HER2-positive breast cancer, with particular emphasis on hybrid E/M states. We further sought to elucidate the interactions between these TICs and adipocytes or fibroblasts, including the contribution of exosome-mediated signaling. Moreover, we aimed to validate the functional significance of these interactions in promoting metastasis using in vivo models. Finally, we sought to link these mechanistic insights to clinical outcomes, including metastatic burden and patient prognosis. Collectively, this work addresses critical gaps in the understanding of HER2-positive breast cancer metastasis and provides a foundation for the development of novel precision therapeutic strategies.

## Materials and Methods

### Sample collection and utilization

All samples were derived from consecutive patients with HER2-positive breast cancer who were admitted to Shanghai General Hospital between January 2023 and March 2025. No specific additional inclusion criteria were applied beyond the confirmed HER2-positive status. This study was approved by the Institutional Review Board of Shanghai General Hospital (approval number: 2021SQ203). All patients provided written informed consent prior to sample collection and data use, in compliance with the Declaration of Helsinki. De-identified patient data and anonymized biological samples were used throughout the study to protect patient privacy. Samples were sequentially enrolled and assigned to different analyses based on the study progress and research objectives at the time of collection. Samples were utilized as follows. First, exosomes and nuclei were concurrently extracted from matched fresh tissues from 4 breast cancer patients. Second, spatial transcriptomics and metabolomics analysis was conducted on fresh samples from 6 patients with breast cancer. Third, spatial proteomics and regional characterization were performed on 12 patients’ samples with intact pathological morphology. Fourth, spatial in situ assays were conducted on 21 paired breast cancer samples, consisting of primary tumor lesions and lymph node metastatic foci with cancer embolus niches. Finally, 12 breast cancer samples (successfully processed, excluding tissues unsuitable for subsequent experiments after grinding) were used for experimental verification and mechanistic investigations.

### Simultaneous extraction of exosomes and cell nuclei from the same tissue

Tissue was mixed with 4 °C prechilled homogenization buffer (phosphate-buffered saline [PBS] containing 2 U/μl RNase inhibitor) and homogenized for 10 s to disrupt the tissue. The homogenate was centrifuged at 3,000 ×*g* and 4 °C for 1 min to separate the supernatant and pellet. The supernatant was further centrifuged at >15,000 ×*g* and 4 °C for 3 min, and the resulting supernatant was filtered through a 0.22-μm filter to obtain exosome-containing filtrate, which was purified using the ExoEasy Maxi Kit (Cat. No. 76064, Qiagen). The initial pellet was resuspended in lysis buffer (10 mmol/l Tris-HCl [pH 7.5], 1 mmol/l CaCl_2_, 5 mmol/l NaCl, 0.1% Triton X-100, 0.2% Tween-20, and 0.4 U/0.22 μl RNase inhibitor), filtered through 40-μm and 30-μm cell sieves, and centrifuged at 800 ×*g* and 4 °C for 15 min to collect cell pellets. Pellets were further purified to remove debris, and the resulting nuclei were washed 5 times to achieve high purity. Single-nucleus transcriptome and exosome small RNA sequencing were performed on the isolated nuclei and exosomes, respectively.

### Single-nucleus transcriptome sequencing and quality control

Nuclei pellets were resuspended in PBS containing 1% bovine serum albumin (BSA). After trypan blue staining to assess viability, nuclei concentration was adjusted to 1,000 to 1,200/μl. cDNA libraries were constructed using the 10x Genomics Chromium Next GEM Single Cell 3′ Reagent Kits v3.1 (16 reactions, Cat. No. 1000268) and Chromium Single Cell 3ʹ Library Construction Kit (Cat. No. 1000020), followed by sequencing on the Illumina Nova 6000 platform in PE150 mode.

Library construction, sequencing, and data analysis were performed by OE Biotech Co., Ltd. (Shanghai, China). Raw sequencing data (fastq format) were processed using 10x Genomics’ Cell Ranger [19], which integrates STAR software for alignment to the GRCh38 reference genome (version: GRCh38).

Using Seurat 4.0.0 [20], low-quality cells were filtered based on number of Unique Molecular Identifiers (UMI), number of detected genes, and percentage. Mito: cells with >200 genes, >1,000 UMIs, log10GenesPerUMI >0.7, mitochondrial UMI ratio <5%, and red blood cell gene ratio <5% were retained as high quality. Doublets were removed using Doublet Finder [21] for downstream analysis, yielding metrics such as high-quality cell count, gene count, and genome alignment rate.

### Advanced bioinformatics analysis of single-nucleus transcriptome data

Highly variable genes (HVGs) were identified using Seurat’s FindVariableGenes. Principal component analysis was performed on HVG expression profiles, and batch effects were corrected using the mutual nearest neighbors algorithm. For annotation of the 11 epithelial clusters, a manual gene-selection strategy was used: highest log fold change in differential expression, maximal cluster purity and separability, and strongest known biological relevance, particularly in the context of cancer. Clustering was performed using the Shared Nearest Neighbors algorithm and visualized using Uniform Manifold Approximation and Projection (UMAP). Marker genes for each cell cluster were identified using Seurat’s FindAllMarkers and visualized with violin and feature plots.

Differential genes were screened using FindMarkers with thresholds: *P* < 0.05 and fold change >1.5. Gene Ontology (GO) and Kyoto Encyclopedia of Genes and Genomes (KEGG) enrichment analyses were performed via hypergeometric tests. The GO terms related to exosome formation and secretion were obtained from the GO database (http://amigo.geneontology.org/amigo/search/ontology?q=exosome).

Cell differentiation trajectories were inferred using Monocle2 (v2.9.0) [22]. Seurat objects were converted to cell data set objects, and order genes (*q* value < 0.01) were selected. Dimensionality reduction and trajectory inference were performed using default parameters.

Ligand–receptor pairs were identified using CellPhoneDB v3.0 [23]. Genes expressed in ≥10% of cells per cluster were analyzed, with 1,000 permutations to generate null distributions. Interaction networks were visualized using IGraph and Circlize software. Integrative analysis was performed using ARACNe (Algorithm for the Reconstruction of Accurate Cellular Networks) and VIPER (Virtual Inference of Protein-activity by Enriched Regulon analysis) to identify distinct transcription factor activity. To build a regulatory network by using ARACNe, 500 PROM1^+^SMAD5^+^ and PROM1^+^SMAD5^−^ cells were randomly sampled as input, respectively. Then, the protein activity based on the combined regulatory network and gene expression signature was inferred by msviper (viper package, version = 1.40.0), setting the maximum size of the regulons as 50 and the number of permutations as 500. Protein–protein interaction (PPI) network analysis—a technique used to explore physical interactions between proteins and identify functional regulatory relationships—was conducted to identify the gene’s regulated targets.

KEGG gene sets (downloaded via Gene Set Enrichment Analysis [GSEA] Base v1.44.0) were used to score pathway activity in individual cells using gene set variation analysis v1.30.0 [24]. Differential pathway activity between groups was calculated using LIMMA v3.38.3 (https://bioconductor.org/help/search/index.html?search-bar=v3.38.3/).

### Exosome small RNA sequencing and analysis

Total RNA was extracted from exosomes using the miRNeasy Serum/Plasma Kit (Cat. No. 217184, Qiagen). RNA quality was assessed via NanoDrop 2000 and an Agilent 2100 Bioanalyzer. Small RNA libraries were constructed from 1 μg of RNA using the NEB Next Small RNA Library Prep Set for Illumina (Cat. No. NEB#E7330S, NEB), followed by sequencing on the Illumina Novaseq 6000 platform (150-bp paired-end reads).

Raw reads were filtered to remove low-quality sequences [e.g., <15 nt, >41 nt, or those containing adapters/poly(A) tails]. Bowtie [25] aligned reads to the Rfam v10.1 [26], RepBase [27], and miRBase [28] databases to annotate noncoding RNAs and known miRNAs. Differentially expressed miRNAs were identified using |log2FC| >1 and *q* value < 0.05. Target genes were predicted via Miranda [29] (*S* ≥ 150, Δ*G* ≤ −30 kcal/mol) and subjected to GO/KEGG enrichment analyses.

### Integrated analysis of single-nucleus transcriptome and exosome small RNA data

Analyses included the following: (a) negative correlation between differential miRNAs and top 10 cell subtype markers (*P* ≤ 0.05, correlation coefficient < −0.3); (b) overlap of coexpressed miRNA–gene pairs with predicted miRNA targets; and (c) visualization via Sankey diagrams, heatmaps, and networks.

### Spatial transcriptomics

StSME (implemented in stLearn [30]) integrates gene expression with tissue morphology and spatial location. Stained images were segmented, and ResNet50-derived morphological features (top 50 PCs) were used to normalize gene expression via weighted averaging (morphological distance as weights). Clustering combines global gene expression with local spatial density.

Pseudo-spatial-temporal (PST) [31–33] reconstructs transcriptional trajectories using spatial and transcriptomic data. Partition-based Graph Abstraction identifies subpopulation connections, and Diffusion Pseudo-time calculates pseudotime. PST distance combines gene expression and physical distance to optimize trajectory graphs.

Spatial neighbor analysis was performed to assess the spatial proximity between distinct cell subpopulations. For each cell, its Euclidean distance to all other cells was calculated based on their 2-dimensional spatial coordinates obtained from imaging datasets. Cells were defined as spatially adjacent neighbors if their Euclidean distance fell below a predefined threshold determined by the average cell diameter in the tissue section. CytoMAP (v1.0), an open-source software tailored for spatial tissue analysis, was used to analyze the fluorescent labels and *x*, *y* coordinates of individual cells within the panorama to identify the spatial distribution of cell neighborhoods and annotate the regions.

Robust cell type decomposition (RCTD), a computational deconvolution algorithm for spatial transcriptomics, was applied to decompose the cell-type composition of each spatial spot. Briefly, reference cell type profiles were derived from relevant single-cell RNA sequencing (scRNA-seq) datasets, and RCTD was performed with default parameters to correct for platform effects between sequencing technologies, thereby accurately estimating the proportion of each cell type in each spot.

### Spatial metabolomics

MS-grade acetonitrile (047138-K2, Thermo Fisher Scientific), formic acid (6450, Thermo Fisher Scientific), and hematoxylin and eosin (H&E) stains (H9627, E4009, Sigma-Aldrich) were used for sample preparation, metabolite extraction, and histological staining. Instruments included the Leica CM 1950 cryostat, airflow-assisted desorption electrospray ionization mass spectrometry imaging (AFADESI-MSI) platform, and the Q-Orbitrap mass spectrometer.

Frozen tissue sections with 10-μm thickness were mounted on charged slides, dried, and adjacent sections were stained with H&E. AFADESI-MSI acquired data in positive/negative ion modes (solvent: 80% acetonitrile, flow rate 5 μl/min). Raw data (.raw) were converted into .imzML via imzML Converter [34], processed in MSiReader (with Cardinal [35] for background subtraction), and normalized by total ion count [36]. Differential metabolites were identified using orthogonal partial least squares discriminant analysis (OPLS-DA) (variable importance in the projection > 1) and *t* tests (*P* < 0.05). Dimensionality reduction (t-distributed Stochastic Neighbor Embedding/UMAP) and spatial shrunken centroids clustering (SSCC) were applied to metabolites annotated using pySM [37] and SmetDB.

### Integration of spatial metabolomics and transcriptomics

Spatial data from both transcriptomic barcodes and metabolomic profiles were translated into a single, standardized spatial marker. Metabolomic pixel data were then matched to their corresponding transcriptomic spots, and the underlying pixel data for each spot were compiled to generate metabolomic datasets specific to each transcriptomic spot.

### Spatial proteomics

For spatial image analysis, Phenochart (v1.2.0; Akoya Biosciences, Inc., Marlborough, MA, USA) was used to define regions of interest (ROIs). Multispectral image cell segmentation and cell phenotyping were accomplished using inForm software (v2.6; Akoya Biosciences, Inc., Marlborough, MA, USA) and QuPath [38]. During this workflow, cell phenotype labels and centroid spatial coordinates of all cells were extracted in parallel. PhenoptrReports (v0.3.2; Akoya Biosciences, Inc., Marlborough, MA, USA) was utilized to integrate processed spatial data and visualize comprehensive cell spatial relationships. After systematic cellular phenotype annotation, cellular neighborhoods with a 50-μm radius were clustered for subsequent spatial profiling. The interactions between neighborhoods were further explored via heatmaps and network analyses.

### Spatial in situ sequencing

Twenty-one pairs of tissue samples from 21 HER2-positive breast cancer patients with lymph node metastasis were selected, and typical embolic cancer cell clusters in primary tumors and lymph node regions were targeted for spatial in situ sequencing using the 10x Xenium platform (10x Genomics), following the manufacturer’s instructions.

Formalin-fixed and paraffin-embedded tissue sections containing target cell clusters were prepared, mounted on 10x Xenium slides, and subjected to deparaffinization and decrosslinking. A custom gene panel was used for padlock probe hybridization, followed by probe ligation, rolling circle amplification (RCA), and automatic on-instrument detection/decoding.

Imaging data were captured by the 10x Xenium analyzer, and the onboard Xenium Onboard Analysis (XOA) pipeline was used for data processing and quality control. Spatial gene expression data were visualized and analyzed using 10x Xenium Explorer software to determine the distribution of target cell subpopulations and their spatial relationships.

### In vitro and in vivo experimental procedures

Ultra-multiplex immunohistochemistry (IHC): Opal 6-Plex staining (AKOYA) was performed on formalin-fixed paraffin-embedded sections. Staining cycles included antigen retrieval, antibody incubation, and opal labeling. Slides were scanned using Vectra Polaris.

Single-cell isolation: Tissues were digested (Human Tumor Dissociation Kit, Miltenyi Biotec), filtered (70 μm), and centrifuged at 300 ×*g* and 4 °C for 5 min to pellet the cells. Red blood cells were lysed if necessary. Dead cells were removed (Dead Cell Removal Kit, Miltenyi Biotec). Intracellular staining was performed using fixation/perm buffers, with compensation adjusted via UltraComp eBeads. Samples were analyzed on a BD Fortessa.

Proximity ligation assay (PLA): PLA was employed after integrating and refining our previous experimental protocols as follows. These 2 subpopulations of cells, cultured on coverslips, were assessed using the Duolink In Situ Red Starter Kit Mouse/Rabbit for PLA. The process involved fixing, permeabilizing, and blocking the slides, followed by staining for epidermal growth factor receptor (EGFR) and coatomer protein complex subunit alpha (COPA). After staining, the slides were processed through PLA probe incubation, ligation, and amplification steps. Finally, the slides were photographed after being mounted with Duolink In Situ Mounting Medium, which includes 4′,6-diamidino-2-phenylindole, dilactate (DAPI) to stain the cell nuclei.

Coculture system and treatment: To explore the effect of DCN^+^TM4SF1^+^ or ADIPOQ^+^NOTCH4^+^ cells on PROM1^+^SMAD5^+^ cells, DCN^+^TM4SF1^+^ or ADIPOQ^+^NOTCH4^+^ cells were sorted and seeded into the upper chamber, while PROM1^+^SMAD5^+^ cells were placed in the lower chamber of 24- or 6-well cell culture inserts with a 0.4-μm pore membrane. The 2 cell types were cocultured at a 1:1 ratio. For 3-dimensional (3D) culture and fluorescence-related assays, 24-well plates were used at a seeding density of 1 × 10^4^ cells per well. For Western blotting (WB), fluorescence-activated cell sorting (FACS), and in vivo experiments, 6-well plates were used at 3 × 10^5^ cells per well. Selected coculture groups were treated with COPA-specific neutralizing antibody at 1 μg/ml for 48 h before subsequent experimental procedures.

EGFR inhibitor (erlotinib) treatment assay: PROM1^+^SMAD5^+^ cells were treated with 1 μmol/l erlotinib (CP-358774, Sellect) for 4 h, followed by WB detection of related proteins.

SMAD5 knockdown assay using shRNA: Lipofectamine RNAiMAX was used for siRNA transfection. PROM1^+^SMAD5^+^ cells were seeded at a density of 1 × 10^5^ cells/well in a 6-well culture plate overnight and culture medium was then changed to Opti-MEM. The transfection system was prepared as follows: 9 μl of Lipofectamine RNAiMAX diluted in 150 μl of Opti-MEM and then mixed with siRNA-Opti-MEM. After 5 min of mixing, the transfection system was then added to wells and interference was carried out for 48 h.

WB analysis: The protein used for WB was derived from the supernatant or cell lysate. Cell culture medium was concentrated by Amicon Ultra-4 Centrifugal Filter Devices (10 kDa MWCO, Cat. No. UFC8010D, Merck Millipore) after centrifugation at 7,500 ×*g* and 4 °C for 40 min. The condensed samples (approximately 200 μl) were mixed with 5× loading buffer, boiled for 3 min, and then analyzed by WB. Cell lysates were obtained after coculture for 48 h. The protein was separated on a sodium dodecyl sulfate-polyacrylamide gel electrophoresis, transferred to polyvinylidene fluoride membranes, and blocked with 5% nonfat dry milk in tris-buffered saline with Tween-20 (TBST). After washing 3 times with TBST, primary antibodies dissolved in antibody buffer were used. A detailed list of all antibodies and related reagents is provided in Table S1.

Cell viability test: A total of 3,000 cells were seeded in 96-well plates. The next day, the cells were treated with a 5-fold gradient of taxol (concentration range of 0.64 to 2,000 μmol/l). After 48 h, 10 μl of CCK-8 was added per well. After 4 h, the absorbance value at a wavelength of 490 nm was detected with a microplate reader. The survival ratio was calculated using the following formula: Survival ratio = (OD_taxol_ − OD_blank_)/(OD_DMSO_ − OD_blank_). The 50% inhibitory concentration (IC_50_) was calculated using GraphPad Prism. Table S2 contains other materials, experimental kits, instruments, and software.

Apoptosis assay (Annexin V/propidium iodide [PI]): Cells were treated with 10 μmol/l taxol for 48 h. Cells were harvested and resuspended with 100 μl of Annexin V binding buffer and incubated at 20 to 25 °C for 5 min. Next, 5 μl of AnnexinV-FITC (V13242, Molecular Probes) and 5 μl of PI (V13242, Molecular Probes) were added, mixed, and incubated at room temperature for 15 min, avoiding light. For flow cytometry analysis, cells were subjected to FACS Calibur flow cytometer (BD Biosciences, San Jose, CA, USA) and analyzed with WinMDI vers. 2.9 software.

Electron microscopy analysis of exosomes: Exosomes were diluted to an appropriate concentration, and 10 μl was carefully dropped onto a copper grid and incubated for 10 min. Excess liquid was removed. Subsequently, 10 μl of 2% uranyl acetate was applied to the grid and incubated for 3 min, followed by removal of excess stain. The grid was air-dried naturally, and transmission electron microscopy was performed at 100 kV for imaging.

Exosome isolation and processing: Cell culture supernatants were collected and centrifuged at 300 ×*g* and 4 °C for 10 min. The supernatant was transferred and centrifuged at 2,000 ×*g* and 4 °C for 20 min, and the resulting supernatant was transferred to a new tube. A 10-kDa ultrafiltration tube (pretreated with ddH₂O by centrifugation at 4,600 ×*g* and 4 °C for 10 min) was used to concentrate the supernatant at 4,600 ×*g* and 4 °C for 20 min. Multiple rounds of concentration were performed until the volume reached approximately 25 ml. The concentrated solution was filtered through a 0.22-μm filter to trap ectosomes on the filter. The filtrate was collected into an ultracentrifuge tube, followed by ultracentrifugation at 150,000 ×*g* and 4 °C for 90 min to pellet exosomes. The supernatant was carefully aspirated, and the pellet was resuspended in 200 μl of PBS, then diluted with 25 ml of PBS and ultracentrifuged again at 150,000 ×*g* and 4 °C for 90 min. The final pellet was resuspended in approximately 100 μl of PBS and adjusted to a stock concentration of 1 μg/μl, aliquoted, and stored at −80 °C. Exosomes were used to treat cells at a final concentration of 10 μg/ml.

miR-671-3p transfection assay: PROM1^+^SMAD5^+^ cells (50% to 60% confluence in 6-well plates) were transfected with miR-671-3p mimic or negative control mimic using liposome reagent (Invitrogen, Thermo Fisher Scientific, USA) as per the manufacturer’s instructions. After 48 h, cells were collected, and Claudin1 mRNA level was detected by quantitative real-time polymerase chain reaction (qRT-PCR). Table S3 lists all plasmids, siRNA and related nucleic acid sequences.

3D spheroid assay: Rat tail collagen type I was diluted to 50 μg/ml with 0.02 N acetic acid. A 500-μl volume was added to each well of a 24-well plate and incubated at room temperature for 1 h. The remaining solution was carefully aspirated, and the wells were rinsed 2 to 3 times with PBS to remove residual acid. Cells were resuspended in an appropriate volume of RPMI 1640 medium to prepare a serial gradient of cell concentrations with a final volume of 500 μl per tube. A second tube was prepared containing RPMI 1640 medium, collagen, and NaOH at a ratio of 5:1:0.0235. The 2 solutions were mixed thoroughly on ice before plating. Following 1 week of incubation, spheroids were counted under a phase-contrast microscope (CKX53, Olympus, Tokyo, Japan).

Metastasis functional assay: Matrigel was thawed slowly at 4 °C for at least 24 h and maintained on ice throughout. It was diluted 1:9 (v/v) with prechilled serum-free medium, and 50 to 100 μl of the diluted gel was added to the upper Transwell chamber to evenly coat the membrane without bubbles. The plate was incubated at 37 °C for 2 h to allow gel solidification, followed by rehydration with 100 μl of serum-free medium at 37 °C for 30 min prior to use; excess medium was discarded afterwards. Cell pellets were washed twice with PBS to eliminate serum interference, then resuspended in BSA-containing serum-free medium at a density of 5 × 10^4^ cells/ml. Next, 700 μl of complete medium with 10% fetal bovine serum (FBS) was added to the lower chamber of a 24-well plate, and Transwell inserts were placed carefully to avoid air bubbles under the membrane. A total of 200 μl of the prepared cell suspension was then added to the upper chamber.

Surface plasmon resonance (SPR): SPR was performed to assess the interaction between EGFR and COPA proteins via amino coupling-based immobilization, using 1.0× PBS-P^+^ (pH 7.4) as the coupling buffer and 1.0× PBS-P^+^ (pH 7.4) supplemented with 5% (v/v) dimethyl sulfoxide as the interaction buffer. A CM5 chip was loaded into the instrument, and channel 2 was activated with EDC/NHS at 10 μl/min. Ligand protein was diluted to 50 μg/ml with sodium acetate and immobilized on the same channel at 10 μl/min, followed by blocking with ethanolamine at 10 μl/min. Analytes were serially diluted and injected from low to high concentrations at 30 μl/min for 60 s; the chip was regenerated with 10 mM glycine-HCl (pH 2.0) for 5 min after each concentration run. Data were globally fitted to a 1:1 Langmuir binding model using Biacore Insight Evaluation software (Cytiva, Uppsala, Sweden; https://www.cytivalifesciences.com.cn/zh/cn/support/software/biacore-downloads/Biacore-S200-Software) to calculate binding and dissociation constants.

Cell transfection and luciferase reporter assay: Experimental verification of the binding between miR-671-3p and the 3′UTR of Claudin1 mRNA. A reporter plasmid containing the wild-type Claudin1 mRNA 3′UTR (predicted to contain the miR-671-3p binding site) and a control plasmid containing the mutant binding site were constructed. These plasmids were cotransfected into PROM1^+^SMAD5^+^ cells together with mimic miR-671-3p or anti-miR-671-3p using Lipofectamine 3000 reagent. Dual-luciferase activity was quantified 48 h after plasmid transfection using the Dual-Luciferase Reporter Assay Kit (Vazyme), strictly following the manufacturer’s instructions. Firefly luciferase signals were normalized to Renilla luciferase values. Data from at least 3 independent experiments were analyzed using 2-way ANOVA to assess the effects, with *P* < 0.05 considered statistically significant.

Immunofluorescence (IF) and confocal laser scanning microscopy: PROM1^+^SMAD5^+^ cells were sorted from HER2-positive breast cancer patients and immediately seeded into Chamber slides containing confocal imaging coverslips at the bottom. After 4 h of culture to allow cell adhesion, concentrated supernatants from different stromal cells were added dropwise, followed by 4 h of incubation. Cells were immediately fixed with ice-cold acetone; after blocking, primary and secondary antibody incubations were performed, each controlled at 1 h and 4 °C to minimize endocytosis and capture the interface of the 2 proteins’ interaction. Confocal microscopy was performed with a Nikon N1, and images were processed with a cooled CCD camera and NIS Viewer software.

Animal experimental design: PY8119 breast cancer cells at low passages (passages 3 to 6) were inoculated into the mouse mammary fat pad at a density of 5 × 10^4^ cells per fat pad, a model in which distant pulmonary metastasis develops. The PY8119 cell line was purchased from Zhongqiao Xinzhou Biotechnology Co., Ltd. The company originally obtained this cell strain from American Type Culture Collection with the catalog number CRL-3278. Prior to experimental use, the cell line was strictly authenticated and verified. Cell identification was performed via short tandem repeat (STR) genotyping by the provider before delivery. For routine maintenance, PY8119 cells were cultured in Ham’s F-12K medium supplemented with 10% FBS and 1% penicillin–streptomycin mixture. Cells were incubated in a humidified incubator with 5% CO₂ at 37 °C and passaged regularly upon reaching 80% to 90% confluence to maintain cell activity and phenotypic stability. Tumors were allowed to grow to the logarithmic phase. Tumors were ground, and cell sorting was performed to isolate PROM1^+^SMAD5^+^, DCN^+^TM4SF1^+^, and ADIPOQ^+^NOTCH4^+^ cells. Coculture systems were constructed, each group was inoculated into the mouse mammary gland in situ, after which tumor growth rates were monitored and compared. When minimal pulmonary metastasis developed (Day 23 according preliminary experiments), tumors were excised, wounds were sutured, and pulmonary metastasis was assessed.

Parabiosis surgery: CD45.1 and CD45.2 mice (female, 8 to 10 weeks, age-matched, obtained from Cyagen Biosciences) were anesthetized with isoflurane (initial 2, adjusted to 1.5 post-anesthesia; water column 0.7) and given erythromycin eye ointment for protection. Their unilateral skin (elbow to knee) was shaved, disinfected with iodine and 70% alcohol, and incised along the flank (proximal knee to elbow, avoiding underlying muscles). The triceps and quadriceps were sutured with 2 interrupted sutures each; flank body walls with 7 to 9 continuous sutures; and skin with 4.0 polypropylene interrupted sutures (absorbable for inner, nonabsorbable for outer), all completed within 15 min. After surgery, mice were placed on a heating pad to wake up, intraperitoneally injected with 200 μl of saline-prepared antibiotics for 3 consecutive days, housed separately, and provided with jelly food due to limited limb movement.

Selected/multiple reaction monitoring analysis of targeted metabolite N-desmethyltamoxifen: (a) Metabolite extraction. Cell samples were collected from −80 °C storage, mixed with prechilled methanol/acetonitrile/water (2:2:1, v/v/v), and vortexed and ultrasonicated for 30 min. The supernatant was harvested, vacuum-dried, and reconstituted with 200 μl of 50% methanol for liquid chromatography-tandem mass spectrometry (LC-MS/MS) analysis. (b) Chromatographic and mass spectrometric analysis. Ultra-high performance LC conditions—Separation was performed on a Shimadzu Nexera X2 LC-30AD system. Mobile phases were water (A) and acetonitrile with 0.1% formic acid (B). Samples were kept at 4 °C; column temperature, 40 °C; flow rate, 300 μl/min; injection volume, 1 μl. Gradient: 0 to 4 min, B from 50% to 90%; 4 to 6 min, B held at 90%; 6 to 7 min, B back to 50%; and 7 to 10 min, B maintained at 50%. MS conditions—Analysis was run on a 5500 QTRAP mass spectrometer in ESI+ mode. Source parameters: 550 °C, GS1 55, GS2 55, CUR 35, IS 5,500 V. MRM mode was used, with ion transitions for N-desmethyltamoxifen as 358.3 > 72* (quantitative) and 358.3 > 129 (qualitative). (c) Data processing. Peak areas and retention times were extracted via Analyst 1.6.3 software (SCIEX, Concord, Ontario, Canada; https://sciex.com). Qualitative and quantitative analysis was conducted based on standard references, with peak areas indicating relative metabolite content. Final absolute quantification results were obtained using linear regression equations.

Tumors were graded using Elston–Ellis criteria [39]. All histological and IHC tumor slides were evaluated by 2 pathologists. Histological grades and all biological features were evaluated based on the invasive components. The cutoff for estrogen receptor and progesterone receptor positivity was 1% positive for tumor cells with nuclear staining. Positivity for HER2 was either IHC HER2 3+ or fluorescence in situ hybridization amplified (ratio of HER2 to Centromere Enumeration Probe 17 of ≥2.0 or average HER2 copy number ≥6.0 signals/cell). The Ki-67 index was expressed as the percentage of positively nuclear staining cells among at least 1,000 invasive cells in the scored area. Survival analyses were performed using log-rank tests and Cox regression analyses (SPSS 17.0). Group comparisons were conducted using *t* tests, analysis of variance (ANOVA), or chi-square tests (*P* < 0.05).

### Statistical analyses

Continuous data were presented as mean ± standard error of the mean after verifying normal distribution. One-way ANOVA was used to compare cytokine levels across different pathological groups. All statistical analyses were conducted using IBM SPSS Statistics 23.0 and GraphPad Prism 7.0, with a 2-tailed *P* value < 0.05 defined as statistically significant.

## Results

### Single-nucleus RNA sequencing revealed the presence of PROM1^+^SMAD5^+^ epithelial cells with pEMT characteristics in HER2-positive breast cancer

**Fig. 1.**
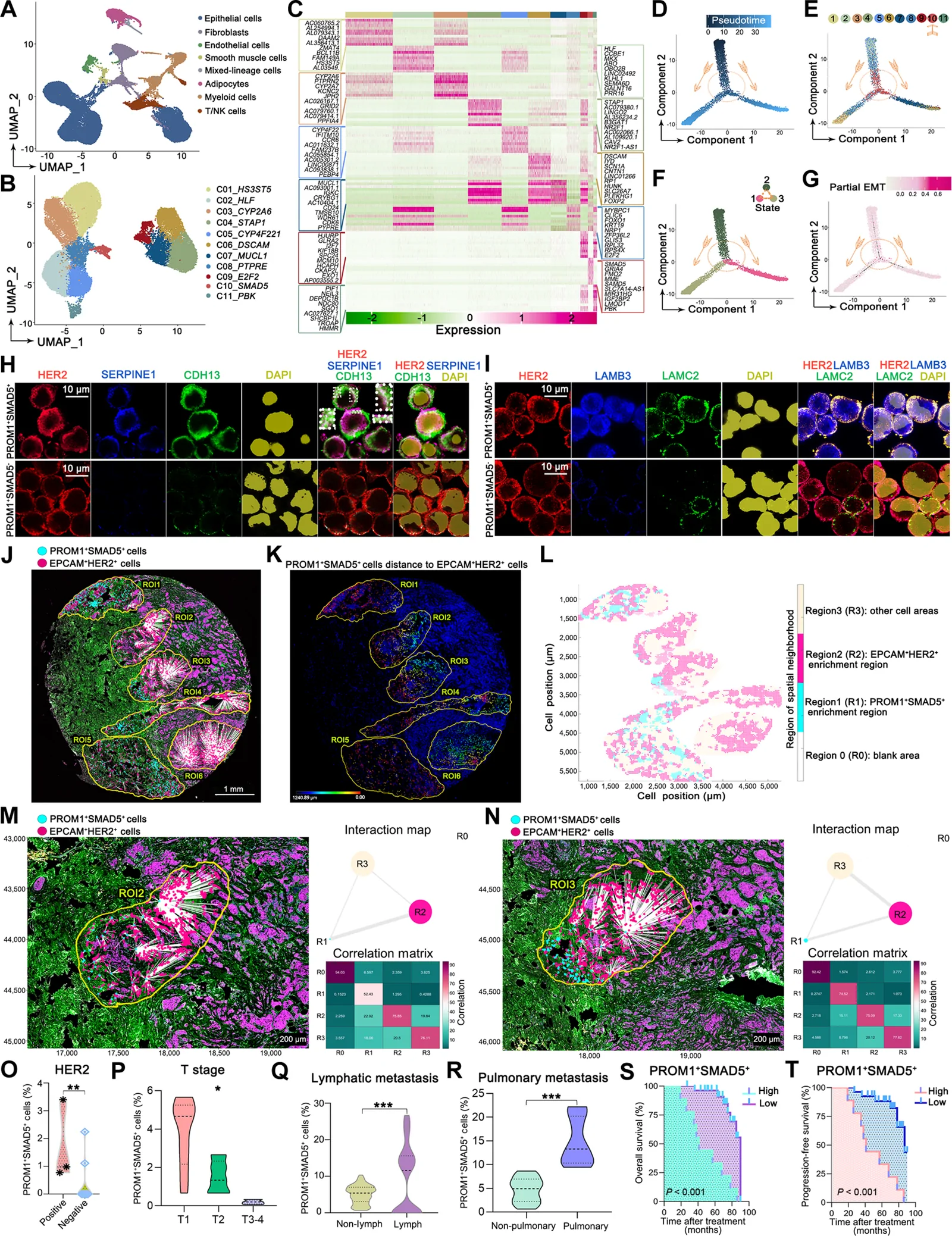
Single-nucleus RNA sequencing (snRNA-seq) revealed the presence of PROM1^+^SMAD5^+^ epithelial cells with partial epithelial–mesenchymal transition (EMT) characteristics in HER2-positive breast cancer. (A) Unsupervised clustering and UMAP visualization display 8 cell types. (B) Epithelial cells were first defined using *EPCAM*, *KRT19*, and *PROM1* markers, then further delineated into 11 distinct subsets. (C) Unsupervised clustering heatmap shows the top 10 genes for each of the 11 subpopulations of epithelial cells. (D to G) Pseudotime plots tracing epithelia cell differentiation (D), subsets dynamics (E), cellular states (F), and pEMT gene signatures (G) along spatiotemporal coordinates. (H and I) In the PROM1^+^SMAD5^+^ and PROM1^+^SMAD5^−^ subpopulation, IF demonstrates pEMT-related markers. (J) The nearest PROM1^+^SMAD5^+^ cells to each EPCAM^+^HER2^+^ cell in multiple ROIs were analyzed using phenoptrReports, and the data were stitched into a complete panorama using Photoshop. ROI1, infiltration of inflammatory cells; ROI2, invasive carcinoma and surrounding stroma; ROI3, invasive carcinoma; ROI4, tumor stroma (left: lymphocyte infiltration; right: blood vessels); ROI5, tumor-adjacent tissue; ROI6, invasive carcinoma. (K) Cell segmentation and analysis of multiple panoramic ROIs were performed using QuPath, including distance-based spatial heatmap analysis of EPCAM^+^HER2^+^ and PROM1^+^SMAD5^+^ cells, with a scale range of 0 to 1240.89 μm (red tones correspond to shorter distances and smaller values, while blue tones correspond to longer distances and larger values). (L) CytoMAP was used to identify the spatial distribution of cell neighborhoods and annotate the regions. (M and N) In ROI 2 and ROI 3, the nearest neighbor distance between each EPCAM^+^HER2^+^ cell and its closest PROM1^+^SMAD5^+^ cell was calculated (left). Region interaction network diagrams, where the size of the nodes indicates the number of elements (cells) within the neighborhood, and the straight lines represent shared boundaries between neighborhoods. The line thickness corresponds to the percentage of shared border, with thicker lines indicating a higher proportion of shared boundaries (upper right). Region interaction heatmaps showing specific values for the interactions between different regions (lower right). Correlation analysis of PROM1^+^SMAD5^+^ cell proportion with breast cancer clinicopathological characteristics, including HER2 status (O), histological grade (P), lymph node involvement (Q), pulmonary metastasis (R). *P < 0.05, **P < 0.01, ***P < 0.001. Correlation of PROM1^+^SMAD5^+^ cell levels with overall survival (S) and progression-free survival (PFS) (T) in breast cancer patients, using the median cell proportion as the cutoff value. Abbreviations: UMAP, Uniform Manifold Approximation and Projection; EMT, epithelial–mesenchymal transition; EPCAM, epithelial cell adhesion molecule; KRT19, keratin 19; PROM1, prominin 1; HER2, human epidermal growth factor receptor 2; SERPINE1, serpin family E member 1; CDH13, cadherin 13; DAPI, 4′,6-diamidino-2-phenylindole, dilactate; LAMC2, laminin gamma 2; LAMB3, laminin beta 3; SMAD5, SMAD family member 5; ROI, region of interest; TNBC, triple-negative breast cancer.

We extracted exosomes from the tumor tissues of 4 patients with HER2-positive breast cancer and simultaneously conducted single-nucleus RNA sequencing (snRNA-seq). Following quality control, 2,294 single nuclei were retained for downstream analyses, with an average of 36,601 genes detected per nucleus. Eight cell types were identified using unsupervised clustering and UMAP visualization (Fig. 1A). The details of the 8 cell types defined by markers are as follows: epithelial cells marked by epithelial cell adhesion molecule (*EPCAM*), keratin 19 (*KRT19*), and prominin 1 (*PROM1*); fibroblasts marked by *DCN* (decorin), *COL1A1* (collagen type I alpha 1 chain), and *COL1A2* (collagen type I alpha 2 chain); adipocytes marked by *ADIPOQ* (adiponectin), *CIDEA* (cell death inducing DFFA-like effector A), and *PLIN1* (perilipin 1); endothelial cells marked by *PECAM1* (platelet and endothelial cell adhesion molecule 1) and *VWF* (von Willebrand factor); smooth muscle cells marked by *ACTA2* (actin alpha 2, smooth muscle), *MYH11* (myosin heavy chain 11), and *MYLK* (myosin light chain kinase); T/NK cells marked by *CD3D* (CD3 delta chain), *CD3E* (CD3 epsilon chain), *CD3G* (CD3 gamma chain), *GNLY* (granulysin), *NCR1* (natural cytotoxicity triggering receptor 1), and *NKG7* (natural killer cell granule protein 7); myeloid cells marked by *CD163* (CD163 molecule), *MRC1* (mannose receptor C-type 1) and *CSF1R* (colony stimulating factor 1 receptor); and mix-lineage cells highly expressed some of the respective markers of both myeloid cells and adipocytes.

Epithelial cells were further divided into 11 distinct subsets (Fig. 1B). For annotation of the 11 epithelial clusters, we used a manual gene-selection strategy. The prioritization criteria included highest log fold change in differential expression, maximal cluster purity and separability, and strongest known biological relevance, particularly in the context of cancer. Unsupervised clustering analysis (Fig. 1C) and GO enrichment analysis (Fig. S1A) suggested that the *C10* subset, which highly expressed *SMAD5*, may be associated with EMT.

Analysis of pseudotime plots demonstrated that with timeline progression (Fig. 1D), the *C10* subset—positioned at the transition initiation points—underwent bidirectional movement (Fig. 1E). This was accompanied by cellular transitions from state 2 to states 1 and 3 (Fig. 1F). When high plasticity and corresponding pEMT gene [40,41] signature scores were mapped to each subset (Fig. 1G), *C10* displayed the highest expression levels of pEMT-associated genes (Fig. 1E), indicating high plasticity within this subset. By comparing with the cell state diagram (Fig. 1F), it can be concluded that the trajectory progresses from cell state 2 to cell states 1 and 3, respectively. Hence, we defined cell state 2 as the prebranch, and cell states 1 and 3 as the 2 branches, and constructed a pseudotime heatmap (Fig. S1B). It can be seen that Module 2 (outlined by a dashed box) represents the transition process from the prebranch to the 2 directions, which is consistent with the trajectory of the subset studied in our research. Therefore, further GO analysis of this module (Fig. S1C) revealed that the biological functions markedly up-regulated in the subset with high SMAD5 expression are closely related to cell movement (outlined by a dashed box).

In the context of our classification of the major epithelial cell categories, the *PROM1* gene was tightly linked to cell stemness. Notably, among all epithelial subpopulations, the proportion of PROM1^+^SMAD5^+^ cells was the highest within the C10 subpopulation (Fig. S2A). To determine whether PROM1^+^SMAD5^+^ cells could serve as a relevant subgroup for further study, we investigated their stemness properties using mammosphere formation (Fig. S2B and C) and limiting dilution transplantation in immunodeficient mice (Fig. S2D to I). These assays support that PROM1^+^SMAD5^+^ cells exhibit stem cell-like characteristics. Therefore, we defined these cells as partial EMT-like epithelial cells.

Consequently, we sorted the PROM1^+^SMAD5^+^ and PROM1^+^SMAD5^−^ subpopulations from HER2-positive breast cancer samples, and compared discrepancies in pEMT-related gene expression between them. Relative to the PROM1^+^SMAD5^−^ subpopulation, serpin family E member 1 (*SERPINE1*), cadherin 13 (*CDH13*), laminin gamma 2 (*LAMC2*), and laminin beta 3 (*LAMB3*) were markedly up-regulated in the PROM1^+^SMAD5^+^ subset (Fig. 1H and I). *SERPINE1* is a key mediator of pEMT [42]; *CDH13* regulates cell adhesion and metastatic potential [43]; and *LAMC2* and *LAMB3* are involved in extracellular matrix remodeling and cancer cell invasion [44,45]. These results indicate a potential regulatory role of *SMAD5* in the acquisition of pEMT characteristics by cells.

For pathological diagnosis, a panoramic spatial analysis was used to characterize the distribution and interactive patterns of PROM1^+^SMAD5^+^ epithelial cells and EPCAM^+^HER2^+^ cancer cells within the pathological TMEs (Fig. 1J). Evaluation of the expression profiles and spatial distances of these 2 subpopulations revealed their enrichment in 3 specific regions: areas with inflammatory cell infiltration, stromal regions with dense blood vessels, and tumor-adjacent tissues. The spatial distance between PROM1^+^SMAD5^+^ epithelial cells and EPCAM^+^HER2^+^ cancer cells was quantified within the QuPath Spatial Analysis module (Fig. 1K). In this visualization, the red points indicate close proximity between the PROM1^+^SMAD5^+^ and EPCAM^+^HER2^+^ cells, whereas blue points denote greater spatial separation. Notably, the shortest distances between the 2 subpopulations were consistently observed within the aforementioned pathological regions. Spatial mapping of all cell populations across the 6 regions, including PROM1^+^SMAD5^+^ cells, EPCAM^+^HER2^+^ cells, and other cell types (others) was performed to visualize their tissue distribution (Fig. 1L). Analysis of inter-regional interaction intensities showed that despite the relatively low proportion of the PROM1^+^SMAD5^+^ subpopulation, its interaction intensity with both EPCAM^+^HER2^+^ and other cells was substantial. This pattern was particularly prominent in ROI2 (invasive carcinoma and surrounding stroma) and ROI3 (invasive carcinoma), both of which correspond to invasive carcinoma (Fig. 1M and N and Fig. S3).

Clinically, PROM1^+^SMAD5^+^ cells were enriched in HER2-positive tumors (Fig. 1O) and more malignant subtypes (Fig. S4A), associating with initial T stage (Fig. S4B). Specifically, we analyzed the expression difference of PROM1^+^SMAD5^+^ cells in 27 HER2-positive patients with different tumor sizes (Fig. 1P); explored the positive percentage of PROM1^+^SMAD5^+^ cells in 248 HER2-positive patients with and without lymph node metastasis (Fig. 1Q), and in 22 patients with and without pulmonary metastasis (Fig. 1R) by multiplex fluorescence IHC technique; and validated the prognosis effect of PROM1^+^SMAD5^+^ cells in 36 HER2-positive patients by overall survival rate as well as progression-free survival (Fig. 1S and T). All these experimental results consistently confirm the significant clinical value of the research subgroup. Our findings suggest that PROM1^+^SMAD5^+^ cells with pEMT characteristics drive tumorigenesis, progression, and metastasis in patients with HER2-positive breast cancer.

To further validate the generalizability of the cell subset we identified, we validated our findings in 2 independent public scRNA-seq datasets: GSE161529 [46], a dataset consisting exclusively of patients with HER2-positive breast cancer; and GSE176078 [32], a dataset covering all major breast cancer subtypes.

In the HER2-positive breast cancer dataset (GSE161529), we first performed unsupervised clustering to define major cell populations and extracted the epithelial cell compartment (highlighted by the dashed circle in Fig. S4C). Reclustering of epithelial cells yielded 5 distinct subsets (Fig. S4D), all of which showed a near-perfect correspondence with the 3 epithelial subsets identified in our study. Importantly, we successfully detected the C10_SMAD5 subset of interest (Fig. S4E). A Sankey plot further confirmed the close association between C10_SMAD5 and Cluster 4 from automated clustering (Fig. S4F). Finally, we projected the expression of *SMAD5* and *PROM1* onto the UMAP embedding. In addition to confirming the presence of *SMAD5/PROM1* double-positive cells, we observed that these double-positive cells were specifically enriched in Cluster 4 (Fig. S4G).

In the pan-breast cancer dataset (GSE176078): We similarly performed global cell clustering and isolated epithelial cells (dashed circle in Fig. S4H). Automated re-clustering identified 9 epithelial subsets (Fig. S4I), which matched well with the 6 epithelial subsets defined in our study, including the C10_SMAD5 subset (Fig. S4J). Projection of *SMAD5* and *PROM1* onto the UMAP plot verified the existence of double-positive cells (Fig. S4K). Critically, these double-positive cells were specifically enriched in patients with HER2-positive breast cancer (Fig. S4L and M). Therefore, despite substantial differences in cellular heterogeneity between the 2 datasets (as reflected in Fig. S4C and H), both datasets robustly reproduced our epithelial cell subsets, particularly the C10_SMAD5 population. Most importantly, the specific enrichment of SMAD5/PROM1 double-positive cells in HER2-positive breast cancer is highly consistent with our bioinformatic analyses and extensive clinical data.

Collectively, this section identifies a unique PROM1^+^SMAD5^+^ epithelial cell subpopulation possessing pEMT and stem cell-like features, which is spatially enriched in the TME and closely correlated with advanced clinical staging, metastasis and poor prognosis in HER2-positive breast cancer, and its existence and characteristics are further verified in multiple independent public datasets.

### Stromal ADIPOQ^+^NOTCH4^+^ adipocytes and DCN^+^TM4SF1^+^ fibroblasts interact with PROM1^+^SMAD5^+^ cells via the COPA–EGFR ligand–receptor axis

**Fig. 2.**
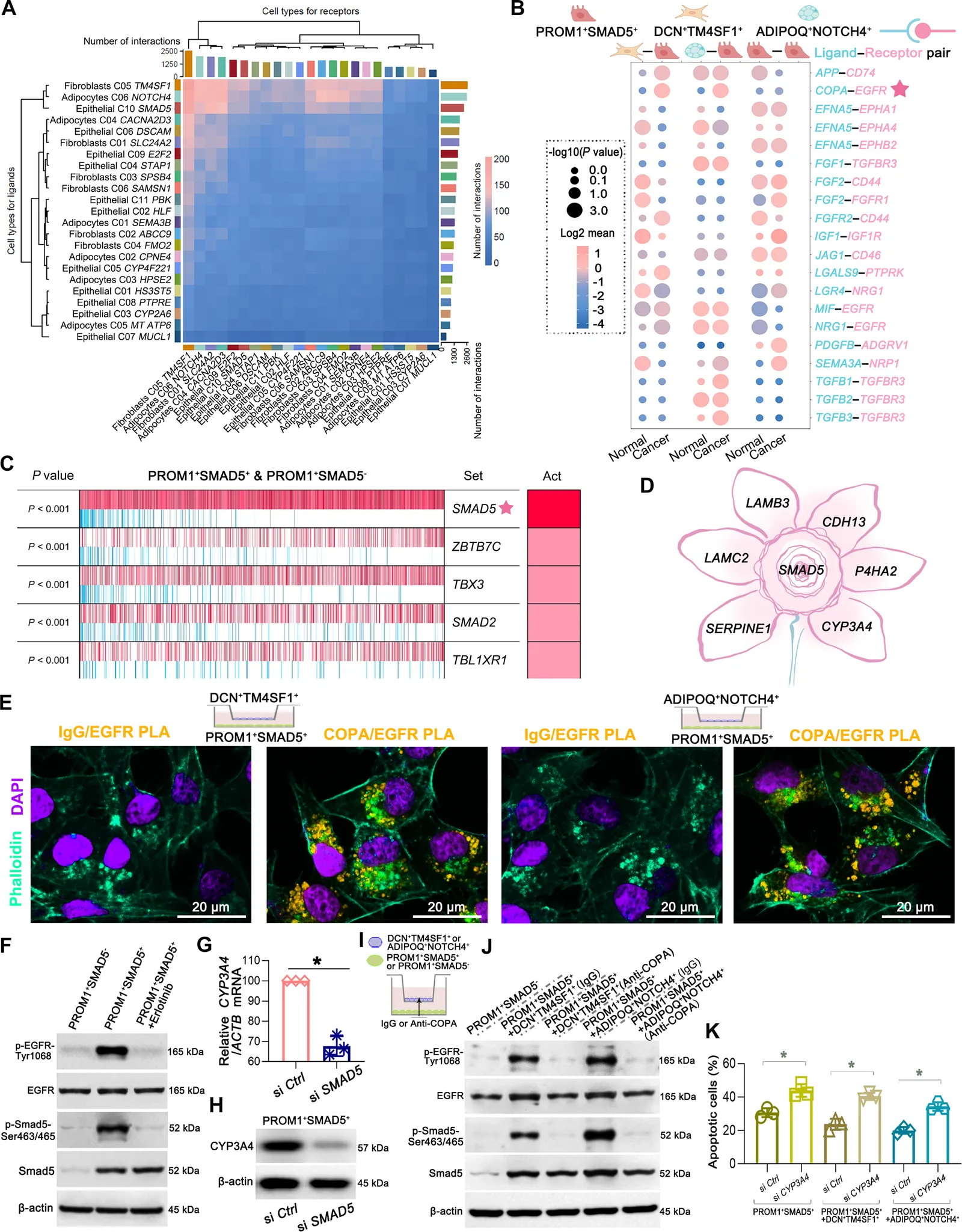
Stromal ADIPOQ^+^NOTCH4^+^ adipocytes and DCN^+^TM4SF1^+^ fibroblasts interact with PROM1^+^SMAD5^+^ cells via the COPA–EGFR ligand–receptor axis. (A) Unsupervised clustering generates cell communication heatmap showing the quantities of ligand–receptor pairs in interactions among epithelial cells, adipocytes, and fibroblasts. (B) CellphoneDB dotplot showing the strength of ligand–receptor pairs in cancer and adjacent normal tissues for epithelial cells (PROM1^+^SMAD5^+^), fibroblasts (DCN^+^TM4SF1^+^), and adipocytes (ADIPOQ^+^NOTCH4^+^). (C) Integrated visualization analysis of the top 5 transcription factors with markedly up-regulated protein activity comparing PROM1^+^SMAD5^+^ to PROM1^+^SMAD5^−^ subpopulations via ARACNe and VIPER. (D) PPI network showing the top SMAD5-regulated target genes associated with pEMT in the PROM1^+^SMAD5^+^ subpopulation. (E) PLA experiment showing the interaction of EGFR and COPA under various coculture conditions and treatments, as indicated in the figure. (F) WB shows the detection of proteins in the cell subpopulations described in the figure legend after the indicated treatments. (G and H) In the PROM1^+^SMAD5^+^ subpopulation, following *SMAD5* gene knockdown, qRT-PCR (G) and WB (H) display changes in the *CYP3A4* gene and protein levels. (I) Schematic representation of the coculture system and experimental setup. (J) WB of cell subpopulations, as described in the figure legend, displaying protein detection after treatment. (K) Apoptosis rate of PROM1^+^SMAD5^+^ cells (treated with 10 μmol/l taxol for 48 h) from indicated monoculture and coculture systems. Cell viability was assessed by CCK8 assay based on OD_450_ values after 48-h treatment. *P < 0.05. Abbreviations: PROM1, prominin 1; SMAD5, SMAD family member 5; ADIPOQ, adiponectin; NOTCH4, notch receptor 4; DCN, decorin; TM4SF1, transmembrane 4 L six family member 1; ARACNe, algorithm for the reconstruction of accurate cellular networks; VIPER, virtual inference of protein-activity by enriched regulon analysis; PPI, protein–protein interaction; PLA, proximity ligation assay; WB, Western blot; EGFR, epidermal growth factor receptor; COPA, coatomer protein complex subunit alpha; CYP3A4, cytochrome P450 family 3 subfamily A member 4; qRT-PCR, quantitative real-time polymerase chain reaction.

As outlined in Background, our study sought to identify the key stromal subpopulations in the TME that influence cancer cells. Unsupervised clustering was used to generate a cell communication heatmap, revealing notable interactions between 2 stromal subsets: C06 adipocytes (high *NOTCH4*) and C05 fibroblasts (high *TM4SF1*), and the C10 epithelial subset (high *SMAD5*) (Fig. 2A). Using cell-type defining markers, we isolated 3 specific cell populations from the dataset: *PROM1^+^SMAD5*^+^ epithelial cells, *ADIPOQ^+^NOTCH4*^+^ adipocytes, and *DCN^+^TM4SF1*^+^ fibroblasts. To screen for relevant ligand–receptor pairs, we applied 3 criteria: (a) *ADIPOQ^+^NOTCH4*^+^ and *DCN^+^TM4SF1*^+^ cells (as ligand sources) must markedly activate *PROM1^+^SMAD5*^+^ cells (as receptors); (b) *PROM1^+^SMAD5*^+^ cells must not activate themselves (to exclude autocrine effects); and (c) activated pairs must be specific to cancer tissues (not adjacent normal tissues). These criteria identified COPA (ligand)–EGFR (receptor) interactions (Fig. 2B).

Integrative analysis with ARACNe and VIPER revealed distinct transcription factor activities between the PROM1^+^SMAD5^+^ and PROM1^+^SMAD5^−^ subpopulations (Fig. 2C). Notably, SMAD5 showed the most pronounced up-regulation in protein activity among all transcription factors (marked with an asterisk). Subsequent PPI network analysis identified *SMAD5*-regulated targets in *PROM1^+^SMAD5*^+^ cells, with cytochrome P450 family 3 subfamily A member 4 (*CYP3A4*) ranking highly alongside pEMT-related genes (Fig. 2D). *CYP3A4* is linked to drug metabolism in multiple cancers, including breast cancer, and plays a key role in drug resistance in malignant tumors [10]. This aligned with our hypothesis that pEMT-characteristic cells may be associated with drug resistance.

First, we sought to determine whether COPA acts as a nonclassical ligand that directly binds EGFR. To verify the direct interaction between COPA and EGFR, we performed SPR assays (Fig. S5A). We purified recombinant human COPA protein and the extracellular domain of human EGFR protein, and SPR analysis confirmed their direct in vitro binding (Fig. S5B).

Next, we examined whether COPA can be secreted into the supernatant and interact with EGFR in *PROM1^+^SMAD5*^+^ cells. As illustrated in the experimental design (Fig. S5C), WB analysis of concentrated stromal cell supernatants showed that COPA was specifically expressed in *DCN^+^TM4SF1*^+^ and *ADIPOQ^+^NOTCH4*^+^ stromal cells (Fig. S5D). Further confocal imaging demonstrated that COPA from the supernatants of *DCN^+^TM4SF1*^+^ and *ADIPOQ^+^NOTCH4*^+^ cells could interact with EGFR in *PROM1^+^SMAD5*^+^ cells (Fig. S5E to H).

Subsequently, we used PLA to confirm the binding between *EGFR* (in *PROM1^+^SMAD5*^+^ cells) and *COPA* (in *DCN^+^TM4SF1^+^* and *ADIPOQ^+^NOTCH4*^+^ cells). Our data support that stromal cells activate the EGFR signaling pathway in adjacent *PROM1^+^SMAD5*^+^ cells in a proximity-dependent manner through secreted COPA protein (Fig. 2E).

Finally, to explore whether EGFR acts as a key cofactor for activating *SMAD5*, we selected an appropriate concentration of the EGFR inhibitor erlotinib to avoid inducing general cellular stress. We used erlotinib at 1 μmol/l, which exerted minimal effects on cell proliferation within 4 to 48 h (Fig. S6A). The stability of *PROM1^+^SMAD5*^+^ cells decreased to below 50% within 48 h (Fig. S6B and C), which sufficiently meets the requirements for subsequent functional experiments.

Subsequently, we sorted *PROM1^+^SMAD5*^+^ and *PROM1^+^SMAD5^−^* subpopulations from breast cancer tissues. In addition to finding that *PROM1^+^SMAD5*^+^ cells could activate *EGFR* and *SMAD5* compared to *PROM1^+^SMAD5^−^* cells, treatment with the EGFR inhibitor erlotinib suppressed the activation of both p-EGFR and p-Smad5 (Fig. 2F and Fig. S6D and E), indicating the activation of the EGFR–SMAD5 signaling axis in *PROM1^+^SMAD5*^+^ cells. Subsequent *SMAD5* knockdown experiments demonstrated reduced *CYP3A4* transcription and protein levels in *PROM1^+^SMAD5*^+^ cells (Fig. 2G and H and Fig. S6F and G), indicating that the EGFR–SMAD5 axis regulates CYP3A4. This supports our hypothesis that stromal subpopulations interact with *PROM1^+^SMAD5^+^* cells via the COPA–EGFR complex, with coupled EGFR–SMAD5 signaling. Coculture experiments (*PROM1^+^SMAD5*^+^ cells with *DCN^+^TM4SF1*^+^ or *ADIPOQ^+^NOTCH4*^+^ cells) combined with IgG/COPA antibody treatment revealed that stromal cells promoted EGFR activation and p-SMAD5 induction in a COPA-dependent manner (Fig. 2I and J and Fig. S6H and I).

Functionally, in both isolated *PROM1^+^SMAD5*^+^ cells and cocultures with *DCN^+^TM4SF1*^+^ or *ADIPOQ^+^NOTCH4*^+^ cells, half-maximum IC_50_ and apoptosis assays were combined to evaluate and characterize the role of CYP3A4 in mediating drug resistance (Fig. 2K and Fig. S6J and K). Collectively, these results indicate that *DCN^+^TM4SF1*^+^ and *ADIPOQ^+^NOTCH4*^+^ cells interact with *EGFR* in *PROM1^+^SMAD5*^+^ cells via COPA, activating the EGFR–SMAD5 axis to up-regulate *CYP3A4* and drive drug resistance.

In summary, stromal ADIPOQ^+^NOTCH4^+^ adipocytes and DCN^+^TM4SF1^+^ fibroblasts secrete COPA to bind EGFR on PROM1^+^SMAD5^+^ cells, activating the downstream EGFR–SMAD5 signaling cascade to up-regulate CYP3A4 expression, which ultimately drives drug resistance in HER2-positive breast cancer cells.

### *DCN^+^TM4SF1*^+^ and *ADIPOQ^+^NOTCH4*^+^ down-regulate *Claudin1* in *PROM1^+^SMAD5*^+^ cells via Exo-miR-671-3p

As described in the first section, we performed parallel single-nucleus and exosome sequencing on the same patients. First, the exosomes underwent quality control assessment, including examination of their characteristic morphology and specific marker expression (Fig. S7A to D). The results confirmed that the quality criteria were satisfied. Next, we performed integrated sequencing analysis.

In the TME, intercellular signaling is not confined solely to typical receptor–ligand binding processes; the transmission of exosomes is a crucial way for cells to communicate [47]. To map the complex communication networks among the 3 main cell types, we designed a comprehensive method that integrated 35 miRNAs with varying expression levels (Fig. S7E) and employed combined analytical techniques (Fig. S7F). This approach consists of 2 mutually reinforcing parts. First, single-sample GSEA was carried out on all cell types and their respective subtypes to determine the enrichment level of exosome-associated GO terms (Fig. S7G). The enrichment scores were displayed in a heatmap, which helped pinpoint cell groups with a stronger ability to secrete exosomes. Second, CellphoneDB analysis was performed on snRNA-seq data to identify ligand–receptor pairs that facilitate interactions between different cells.

**Fig. 3.**
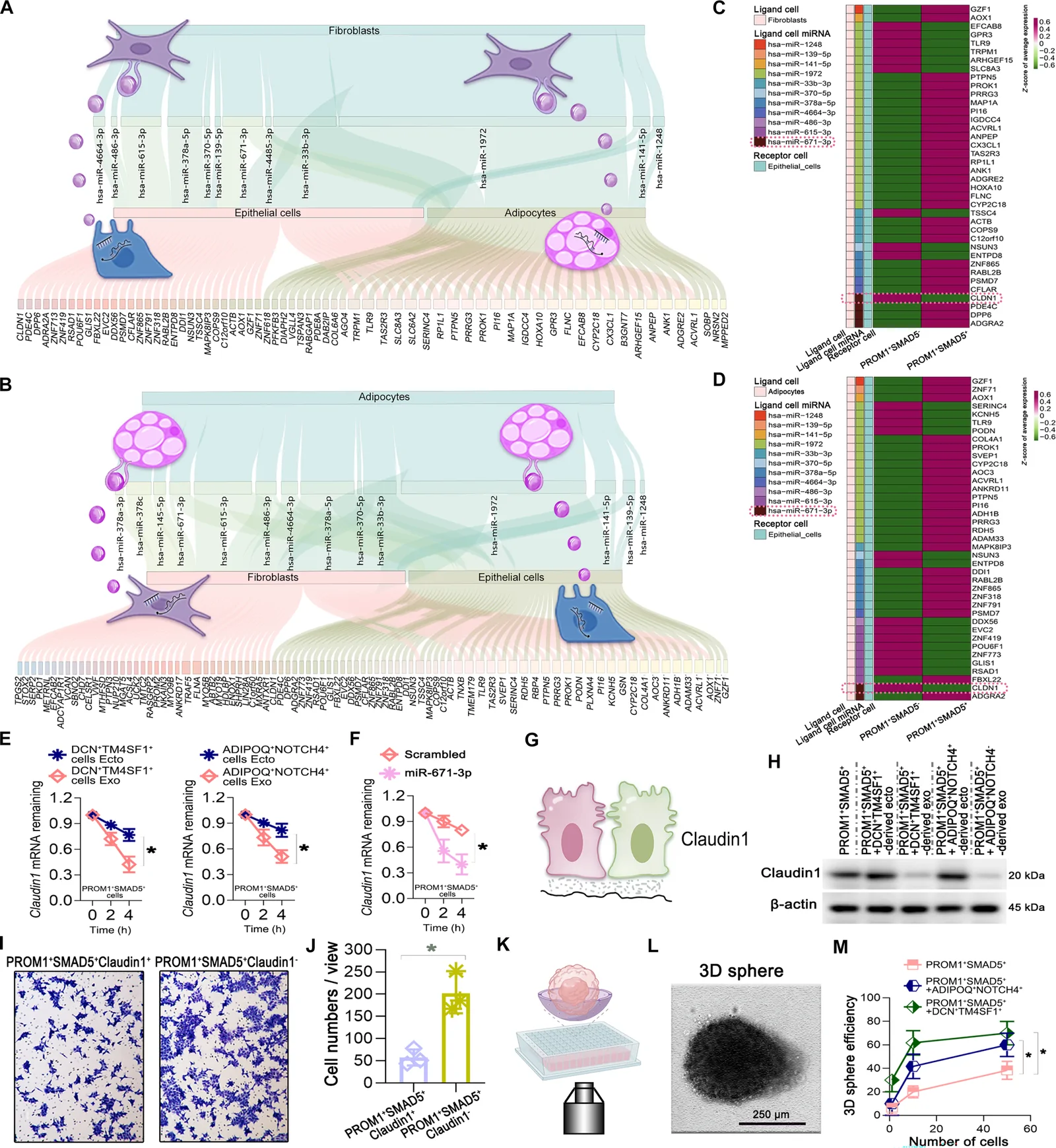
DCN^+^TM4SF1^+^ and ADIPOQ^+^NOTCH4^+^ down-regulate *Claudin1* in PROM1^+^SMAD5^+^ cells via Exo-miR-671-3p. (A and B) Sankey network mapping regulatory interactions: (A) exosomal miRNAs secreted by fibroblasts targeting mRNAs in epithelial cells and adipocytes, respectively; (B) exosomal miRNAs secreted by adipocytes targeting mRNAs in fibroblasts and epithelial cells, respectively. (C and D) Heatmaps demonstrating gene expression changes induced by ligand cell miRNAs derived from fibroblasts (C) and adipocytes (D) targeting receptor cell mRNAs in PROM1^+^SMAD5^−^ vs. PROM1^+^SMAD5^+^ epithelial cells. (E) *Claudin1* RNA stability from PROM1^+^SMAD5^+^ cells quantified by qRT-PCR, after coincubation with DCN^+^TM4SF1^+^-ectosomes or DCN^+^TM4SF1^+^- exosomes, ADIPOQ^+^NOTCH4^+^-ectosomes, or ADIPOQ^+^NOTCH4^+^-exosomes, *P < 0.05. (F) *Claudin1* RNA stability from PROM1^+^SMAD5^+^ cells quantified by qRT-PCR, after coincubation with or without has-miR-671-3p, *P < 0.05. (G) Schematic representation of the role of Claudin1 in the tight junctions of epithelial cells. (H) WB of cell subpopulations, as described in the figure legend, displaying protein detection after treatment. (I) Cell metastasis functional assays in PROM1^+^SMAD5^+^Claudin1^+^ cells and PROM1^+^SMAD5^+^Claudin1^−^ cells. (J) Comparison of cell metastasis ability between the above 2 subpopulations, *P < 0.05. A schematic diagram illustrates spheroid formation in 3D culture plates and interference contrast microscopy observations (K). (L) Phase-contrast microscopy images of 3D spheroid formation and (M) quantification of spheroid numbers, showing differences between groups as indicated in the figure, *P < 0.05. Abbreviations: PROM1, prominin 1; SMAD5, SMAD family member 5; ADIPOQ, adiponectin; NOTCH4, notch receptor 4; DCN, decorin; TM4SF1, transmembrane 4 L six family member 1; miRNA, micro-RNA; WB, Western blot; qRT-PCR, quantitative real-time polymerase chain reaction.

A Sankey network was used to show exosomal miRNAs secreted equally as ligands by fibroblasts and adipocytes, which target the same genes in epithelial recipient cells. These miRNAs and their matched target genes are likely key molecules mediating intercellular communication (Fig. 3A and B). Further analysis identified that exosomal miR-671-3p, secreted by either fibroblasts or adipocytes (as ligand cells), consistently down-regulated the specific target gene *Claudin1* in the *PROM1^+^SMAD5*^+^ subpopulation (Fig. 3C and D).

To verify how exosomes from the 2 cell types regulate *Claudin1* in *PROM1^+^SMAD5*^+^ cells, we extracted exosomes from *DCN^+^TM4SF1*^+^ and *ADIPOQ^+^NOTCH4*^+^ cells separately to treat the *PROM1^+^SMAD5*^+^ subpopulation, using ectosomes as controls. Compared with ectosome-treated *PROM1^+^SMAD5*^+^ cells, their exosome-treated counterparts exhibited reduced mRNA stability of *Claudin1* (Fig. 3E). The direct application of miR-671-3p confirmed its ability to degrade *Claudin1* mRNA (Fig. 3F), suggesting that exosomes from *DCN^+^TM4SF1*^+^ and *ADIPOQ^+^NOTCH4*^+^ cells modulate Claudin1 mRNA stability via miR-671-3p. Claudin family members function via intercellular tight junctions (Fig. 3G) [48]. Down-regulation of *Claudin1* has been linked to enhanced metastasis in multiple cancers, including breast cancer [49], though its role therein remains poorly characterized. Beyond effects on *Claudin1* mRNA stability, exosome treatment (but not ectosome treatment) also reduced Claudin1 protein levels (Fig. 3H and Fig. S7H), indicating that mRNA degradation leads to decreased protein expression.

To verify the regulatory effect of miR-671-3p in exosomes derived from donor cells on Claudin1 mRNA in recipient cells, we performed validation at 3 levels. First, as for the binding of miR-671-3p on the 3′UTR of Claudin1 mRNA, a reporter plasmid containing the wild-type Claudin1 mRNA 3′UTR (predicted to contain the miR-671-3p binding site) and a control plasmid containing the mutant binding site were constructed (Fig. S7I). These plasmids were cotransfected into *PROM1^+^SMAD5*^+^ cells together with mimic miR-671-3p or anti-miR-671-3p. The cotransfection of mimic miR-671-3p significantly inhibited the luciferase activity of the wild-type reporter gene but had no effect on the mutant type, whereas cotransfection of anti-miR-671-3p significantly increased the luciferase activity of the wild-type reporter gene but had no effect on the mutant type (Fig. S7J). These results indicate that miR-671-3p can target Claudin1 mRNA.

Subsequently, from the perspective of inhibiting exosome secretion (Fig. S7K), the exosome secretion inhibitor GW4869 was used to treat donor *DCN^+^TM4SF1*^+^ or *ADIPOQ^+^NOTCH4*^+^ cells, and then these cells were cocultured with recipient *PROM1^+^SMAD5*^+^ cells, respectively, to detect the effect on Claudin1 in *PROM1^+^SMAD5*^+^ cells. Inhibiting the secretion of exosomes derived from *DCN^+^TM4SF1*^+^ or *ADIPOQ^+^NOTCH4*^+^ cells promoted the expression of Claudin1 in *PROM1^+^SMAD5*^+^ cells (Fig. S7L and M), confirming the necessity of exosomes as delivery carriers.

Finally, from the perspective of exosome-specific loss of function, donor *DCN^+^TM4SF1*^+^ or *ADIPOQ^+^NOTCH4*^+^ cells were treated with anti-miR-671-3p or anti-Ctrl, and then their exosomes were isolated (Fig. S7N). These exosomes were used to treat *PROM1^+^SMAD5*^+^ cells, and Claudin1 mRNA was detected. The exosomes pretreated with anti-miR-671-3p lost the ability to degrade Claudin1 mRNA (Fig. S7O), thus proving that the functional miR-671-3p carried by exosomes is necessary for its role in degrading Claudin1 mRNA.

Functionally, *PROM1^+^SMAD5^+^Claudin1*^−^ cells showed higher metastatic potential than *PROM1^+^SMAD5^+^Claudin1*^+^ cells (Fig. 3I and J). Additionally, 3D spheroid assays revealed that coculturing with *DCN^+^TM4SF1*^+^ and *ADIPOQ^+^NOTCH4*^+^ cells further boosted the stemness and spheroid-forming capacity of *PROM1^+^SMAD5*^+^ cells (Fig. 3K to M). Taken together, these results indicate that *Claudin1* down-regulation enhances the metastatic potential and stemness of the *PROM1^+^SMAD5*^+^ subpopulation.

These findings demonstrate that exosomal miR-671-3p derived from the 2 stromal cell populations specifically targets and degrades Claudin1 mRNA in PROM1^+^SMAD5^+^ cells, and the down-regulation of Claudin1 markedly enhances the metastatic potential and stemness of this epithelial subpopulation.

### Contribution of the tripartite interaction among the *PROM1^+^SMAD5*^+^ subpopulation, *DCN^+^TM4SF1*^+^ cells, and *ADIPOQ^+^NOTCH4*^+^ cells to breast cancer metastasis

**Fig. 4.**
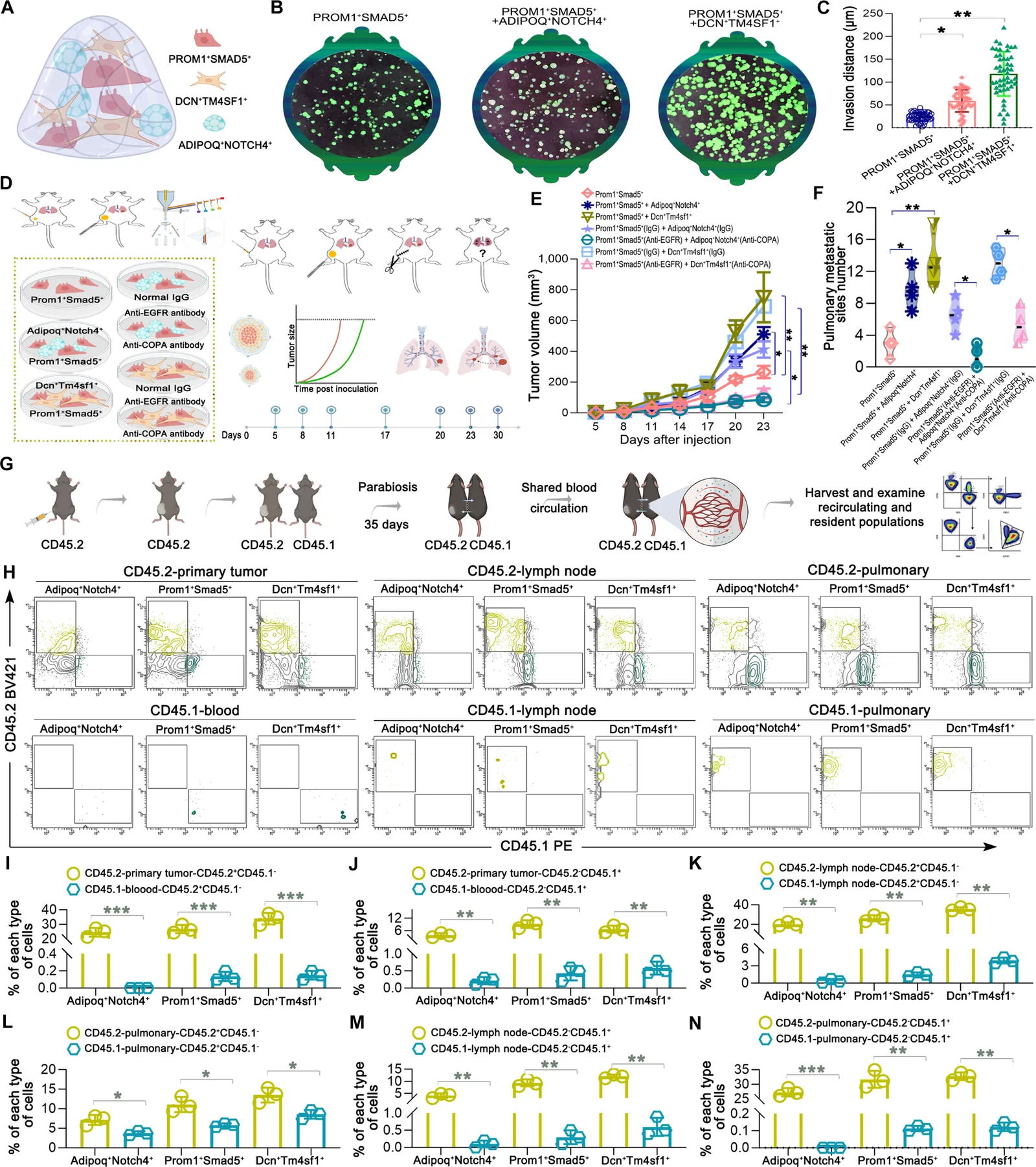
Contribution of the tripartite interaction among PROM1^+^SMAD5^+^ subpopulation, DCN^+^TM4SF1^+^, and ADIPOQ^+^NOTCH4^+^ cells to breast cancer metastasis. (A) Diagram of 3D collagen I invasion assays. PROM1^+^SMAD5^+^ cells were cultured alone or cocultured with stromal cells. (B) Representative confocal images of 3D collagen I invasion assays with GFP-labeled PROM1^+^SMAD5^+^ cells under monoculture and coculture conditions. (C) Quantification of the invasion distances. *P < 0.05, **P < 0.01. (D) Animal experimental design. Differences in mice in situ tumors volume (E) and pulmonary metastatic lesions number (F) in accordance with the treatment conditions illustrated in (D). *P < 0.05, **P < 0.01. (G) Schematic diagram of the experimental design for studying the organ distribution of the 3 subpopulations using the parabiosis model. (H) Flow cytometry showing the distribution percentages of the 3 subpopulations in the indicated organs. (I to N) Quantitative statistical analysis of the flow cytometry results in (I). *P < 0.05, **P < 0.01, ***P < 0.001. Abbreviations: PROM1, prominin 1; SMAD5, SMAD family member 5; ADIPOQ, adiponectin; NOTCH4, notch receptor 4; DCN, decorin; TM4SF1, transmembrane 4 L six family member 1; GFP, green fluorescent protein.

Assessing how *DCN^+^TM4SF1*^+^ and *ADIPOQ^+^NOTCH4*^+^ cells functionally affect the *PROM1^+^SMAD5*^+^ subpopulation, in vitro 3D spheroid assays showed that these 2 stromal subpopulations further enhanced the invasive capacity of *PROM1^+^SMAD5*^+^ cells (Fig. 4A to C).

To investigate the impact of *DCN^+^TM4SF1*^+^ and *ADIPOQ^+^NOTCH4*^+^ cells on *PROM1^+^SMAD5*^+^ epithelial cell-mediated breast tumorigenesis and distant pulmonary metastasis, and the role of the key ligand–receptor pair COPA–EGFR in breast cancer progression and distant pulmonary metastasis, in vivo experiments were performed (Fig. 4D). Coincubation of *PROM1^+^SMAD5*^+^ cells with either *DCN^+^TM4SF1*^+^ cells or *ADIPOQ^+^NOTCH4*^+^ cells significantly enhanced both tumor growth and pulmonary metastasis compared with *PROM1^+^SMAD5*^+^ cells alone, while antibody treatments targeting *COPA* in *DCN^+^TM4SF1*^+^ and *ADIPOQ^+^NOTCH4*^+^ cells, as well as *EGFR* in PROM1^+^SMAD5^+^ cells, resulted in reduced tumor size and fewer metastases (Fig. 4E and F). Collectively, these findings indicate that both stromal subpopulations contribute to tumor initiation and pulmonary metastasis driven by *PROM1^+^SMAD5*^+^ cells. Thus, disrupting ligand–receptor interactions could mitigate these processes and offer potential therapeutic targets for future interventions.

Parabiosis, a classic experimental model involving vascular anastomosis between 2 inbred mice, enables assessment of the tissue retention capacity of target cells. In this study, we investigated whether these 3 focal subpopulations migrate through the circulatory system and reach an equilibrium state between parabionts. We established parabiosis mouse models (Fig. 4G). The gating strategy for flow cytometry first identified *DCN^+^TM4SF1^+^*, *ADIPOQ^+^NOTCH4*^+^, and *PROM1^+^SMAD5*^+^ subpopulations, then analyzed their CD45.1/CD45.2 distribution (Fig. 4H).

The results showed that the proportion of the 3 subpopulations in CD45.2^+^CD45.1^−^ cells from CD45.2 primary tumors was higher than that in CD45.2^+^CD45.1^−^ cells from CD45.1 blood (Fig. 4I), indicating a higher retention probability of these subpopulations in tumors than in blood. Conversely, the proportion of the 3 subpopulations in CD45.2^−^CD45.1^+^ cells from CD45.2 primary tumors was higher than that in CD45.2^-^CD45.1^+^ cells from CD45.1 blood (Fig. 4J), suggesting that the minimal number of these subpopulations derived from CD45.1 mice preferentially enriched in CD45.2 tumors rather than CD45.1 blood. In CD45.2 mice, the proportion of the 3 subpopulations of CD45.2^+^CD45.1^−^ cells from both the lymph nodes (Fig. 4K) and lungs (Fig. 4L) was higher than that in the corresponding tissues of CD45.1 mice, indicating a greater distribution of these subpopulations in metastatic organs of tumor-bearing mice compared with tumor-free mice. In CD45.2 mice, the proportion of the 3 subpopulations in CD45.2^−^CD45.1^+^ cells from both lymph nodes (Fig. 4M) and lungs (Fig. 4N) was higher than that in corresponding tissues of CD45.1 mice, suggesting that the minimal number of these subpopulations derived from CD45.1 mice preferentially enriched in metastatic organs of tumor-bearing CD45.2 mice rather than in the corresponding organs of tumor-free CD45.1 mice. Taken together, these findings indicated that these 3 subpopulations possess tumor-resident characteristics and the ability to metastasize to the lymph nodes and lungs via the bloodstream.

Overall, the tripartite crosstalk between PROM1^+^SMAD5^+^ epithelial cells and the 2 stromal subpopulations collectively promotes tumor growth and pulmonary metastasis in vivo, and the COPA–EGFR ligand–receptor interaction serves as a core mediator of this malignant progression, providing promising targets for antimetastatic intervention.

### Spatial distribution characteristics of *PROM1^+^SMAD5*^+^ and *DCN^+^TM4SF1*^+^ subpopulations in patients with breast cancer and lymph node and pulmonary metastasis

To explore the spatial distribution of *DCN^+^TM4SF1*^+^ and *PROM1^+^SMAD5*^+^ cells, as well as their connections with cancer pathological features, we performed multiplex IHC using 5 analytical systems (Phenochart, inForm, QuPath, phenoptrReports, and CytoMAP) to carry out comprehensive spatiotemporal profiling.

**Fig. 5.**
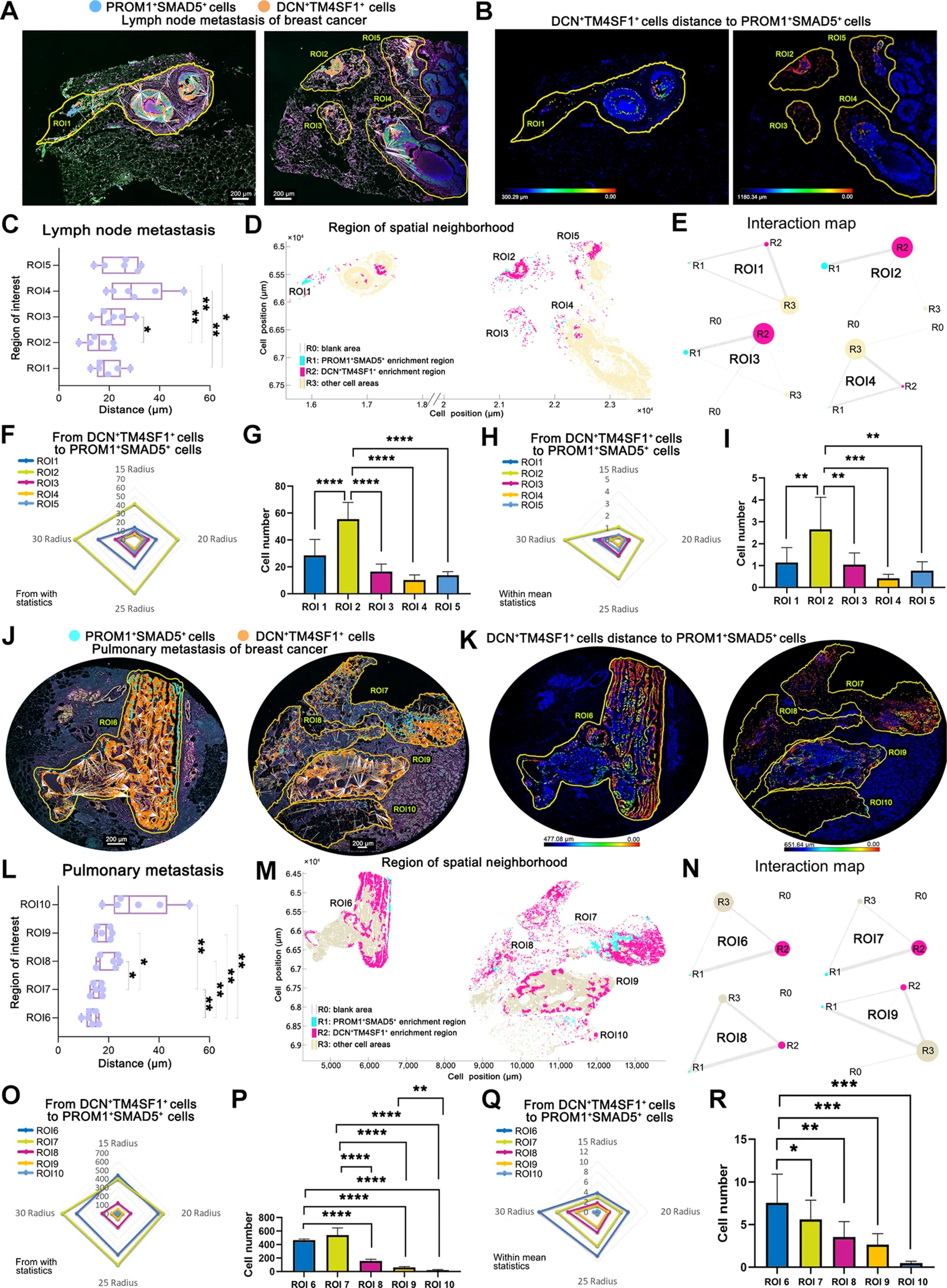
Spatial distribution characteristics of PROM1^+^SMAD5^+^ and DCN^+^TM4SF1^+^ subpopulations in breast cancer patients with lymph node and lung metastasis. (A) PhenoptrReports analyzed the nearest PROM1^+^SMAD5^+^ cells to each DCN^+^TM4SF1^+^ cell in multiple ROIs, with data stitched into a complete panorama of the pathological sections of breast cancer lymph node metastasis. ROI1—duct and necrotic tissue; ROI2—necrotic tissue; ROI3—glandular stroma; ROI4—periductal stroma; ROI5—near the duct. (B) Distance-based spatial heatmap analysis of PROM1^+^SMAD5^+^ and DCN^+^TM4SF1^+^ cells, with scale ranges of 0 to 300.29 μm (left) and 0 to 1,180.34 μm (right). Red denotes shorter distances with smaller values, and blue denotes longer distances with larger values. (C) Comparison of the spatial distance between PROM1^+^SMAD5^+^ cells and stromal DCN^+^TM4SF1^+^ cells among ROI1-ROI5, in 6 cases of breast cancer with lymph node metastasis. *P < 0.05, **P < 0.01. (D) CytoMAP analysis of fluorescent labels and *x* and *y* coordinates of individual cells in multiple ROIs within the panorama to identify the spatial distributions of cell neighborhoods and annotate regions. (E) Region interaction network diagrams, where node size indicates the number of elements (cells) in each neighborhood, and straight lines represent shared boundaries between neighborhoods. The line thickness corresponds to the percentage of shared border, with thicker lines indicating higher proportional overlap. Spatial geography maps reveal the spatial scale between these 2 subpopulations from 2 dimensions: spatial geographic map analysis using “From with” (F) and “Within mean” (H) and respective quantitative statistics (G and I). **P < 0.01, ***P < 0.001, ****P < 0.0001. (J) PhenoptrReports were used to assess the closest PROM1^+^SMAD5^+^ cells relative to each DCN^+^TM4SF1^+^ cell across multiple ROIs, and the resulting data were compiled into a full panoramic view in pathological sections of breast cancer pulmonary metastasis. ROI6, bronchus of the pulmonary; ROI7, most of it is stroma (fibrous tissue, fat, and lymphocytes), with a small number of cancerous components (right corner); ROI8, interstitial (fibrous, adipose); ROI9, pulmonary mucous gland, mucus-secreting salivary gland; ROI10, fat. (K) Spatial heatmap analysis based on distance for PROM1^+^SMAD5^+^ and DCN^+^TM4SF1^+^ cells, with scale ranges of 0 to 477.08 μm (left) and 0 to 651.64 μm (right). (L) Comparison of the spatial distance between PROM1^+^SMAD5^+^ cells and stromal DCN^+^TM4SF1^+^ cells among ROI6 to ROI10, in 6 cases of breast cancer with pulmonary metastasis. *P < 0.05, **P < 0.01. (M) CytoMAP was applied to analyze fluorescent markers and the *x* and *y* coordinates of single cells within multiple ROIs in the panorama, enabling the identification of spatial patterns of cell neighborhoods and region annotation. (N) Diagrams of regional interaction networks, where node dimensions reflect the number of elements (cells) in each neighborhood, and solid lines denote shared margins between neighborhoods. Line thickness is proportional to the percentage of shared borders, with heavier lines signifying greater proportional overlap. Spatial geographic maps illustrate the spatial scale between these 2 subpopulations from 2 perspectives: spatial geographic map analysis utilizing “From with” (O) and “Within mean” (Q), along with their corresponding statistical quantifications (P and R). *P < 0.05, **P < 0.01, ***P < 0.001, ****P < 0.0001. Abbreviations: PROM1, prominin 1; SMAD5, SMAD family member 5; DCN, decorin; TM4SF1, transmembrane 4 L six family member 1; ROI, region of interest.

In the metastatic lymph nodes, 5 key regions were identified based on the distribution of *DCN^+^TM4SF1*^+^ and *PROM1^+^SMAD5*^+^ cells and the degree of their association (Fig. 5A). Analysis of the panoramic spatial distance revealed that the 2 subpopulations were in close proximity to the duct and necrotic tissues (Fig. 5B and C). All *PROM1^+^SMAD5*^+^ cells, *DCN^+^TM4SF1*^+^ cells, and other cell types excluding these 2 subpopulations within the 5 regions were spatially mapped to the tissue, enabling a panoramic view of the spatial distribution and putative cell–cell interaction patterns of the 2 subpopulations within the 5 regions (Fig. 5D). The interaction intensity between PROM1^+^SMAD5^+^ and DCN^+^TM4SF1^+^ cells within ROI2 and ROI3, the invasive carcinoma areas, was stronger than that with other regions (Fig. 5E).

We leveraged the “From with” analysis feature in phenoptrReports, following the software’s analytical procedures to tally the count of “From” cells that have at least one “To” cell within a set radius. From a statistical standpoint, this enabled evaluation of spatial connectivity between the 2 cell phenotypes. In our research, we sought to find out if *DCN^+^TM4SF1*^+^ cells (as “From” cells) are spatially adjacent to *PROM1^+^SMAD5*^+^ cells (as “To” cells). A greater “From with” value means a large number of *DCN^+^TM4SF1*^+^ cells were situated close to *PROM1^+^SMAD5*^+^ cells, implying a spatial link between them, which may indicate that fibroblasts cluster around tumor cells. Conversely, a low value suggests that the 2 cell subpopulations are spatially unrelated. The findings demonstrated that in ROI2 (invasive carcinoma and surrounding stroma), the number of *DCN^+^TM4SF1*^+^ cells surrounding *PROM1^+^SMAD5*^+^ cells was significantly higher than that in other intervals (Fig. 5F and G). This indicated that these 2 subpopulations showed notably different regional enrichment in the necrotic area.

In previous analyses, we used *PROM1^+^SMAD5*^+^ cells as spatial references to examine the relative positioning of *DCN^+^TM4SF1*^+^ cells. To further quantify their spatial association, we used the “Within mean” metric. In our analysis, where *DCN^+^TM4SF1*^+^ cells served as “From” cells and *PROM1^+^SMAD5*^+^ cells served as “To” cells, this metric measured the average number of *PROM1^+^SMAD5*^+^ cells surrounding each *DCN^+^TM4SF1*^+^ cell within a defined radius, thus indicating whether the 2 populations were closely clustered or sparsely distributed. The results demonstrated significantly more *PROM1^+^SMAD5*^+^ cells surrounding *DCN^+^TM4SF1*^+^ cells in ROI2 (invasive carcinoma and surrounding stroma) compared to other intervals (Fig. 5H and I), reinforcing that these 2 subpopulations exhibit region-specific enrichment with marked differences in necrotic areas.

At breast cancer pulmonary metastasis sites, we identified 5 key regions based on the distribution patterns and association strength of *DCN^+^TM4SF1*^+^ and *PROM1^+^SMAD5*^+^ cells (Fig. 5J). Panoramic spatial distance analyses revealed particularly close proximity between the 2 subpopulations in 2 regions: (a) the pulmonary bronchus and (b) a predominantly stromal area (fibrous tissue, fat, and lymphocytes) with minor cancerous components (right corner) (Fig. 5K and L). Spatial mapping of all cell populations within these 5 regions confirmed this pattern, with *PROM1^+^SMAD5*^+^ cells, *DCN^+^TM4SF1*^+^ cells, and other cells showing distinct clustering (Fig. 5M). Inter-regional interaction intensity analysis demonstrated stronger interactions between the metastatic foci and both ROI6 (infiltration of inflammatory cells) and ROI7 (invasive carcinoma and surrounding stroma) than with other regions (Fig. 5N). Consistently, both “From with” (Fig. 5O and P) and “Within mean” (Fig. 5Q and R) analyses confirmed that *PROM1^+^SMAD5*^+^ and *DCN^+^TM4SF1*^+^ cells form tight associations specifically in ROI6 (infiltration of inflammatory cells) and ROI7 (invasive carcinoma and surrounding stroma). Collectively, these findings suggest that the lung bronchus and stromal regions containing fibrous tissue and fat may serve as pioneer niches for metastatic cancer cell colonization.

To conclude, PROM1^+^SMAD5^+^ cells and DCN^+^TM4SF1^+^ fibroblasts exhibit region-specific tight spatial aggregation in metastatic lymph nodes and pulmonary metastatic lesions, and the lung bronchus and stromal-rich areas are confirmed as key niches supporting the colonization of metastatic breast cancer cells.

### Spatial pathological distribution patterns and spatiotemporal differentiation analysis centered on the *PROM1^+^SMAD5*^+^ subpopulation

**Fig. 6.**
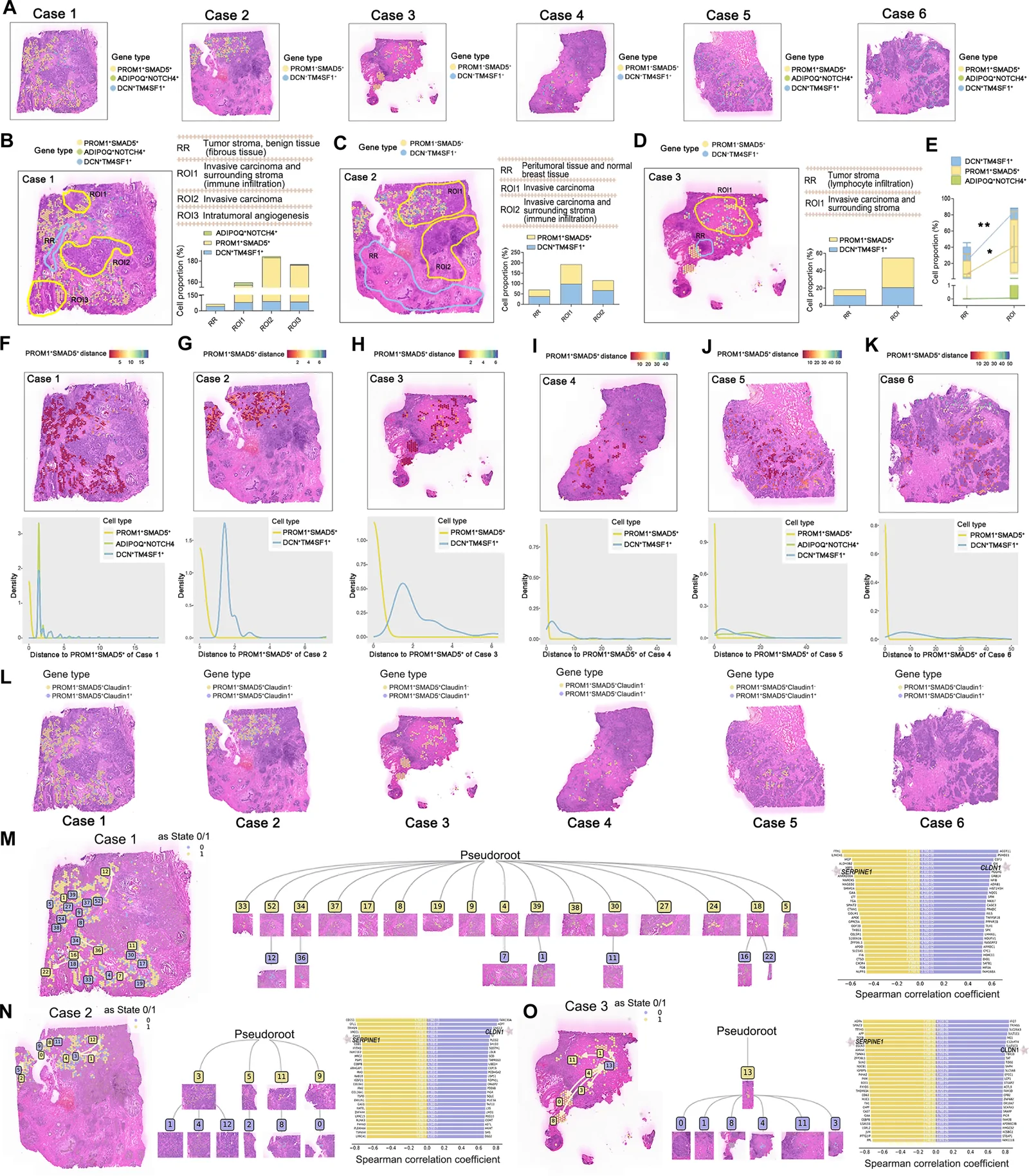
Spatial pathological distribution patterns and spatiotemporal differentiation analysis centered on the PROM1^+^SMAD5^+^ subpopulation. (A) Spatial distribution of the 3 subpopulations or any 2 mentioned above across 6 samples. (B to D) Spatial localization of the 3 (or 2) subpopulations overlaid on H&E staining (left); pathological features of the delineated spatial regions (upper right); quantification of the relative percentages of the subpopulations within the delineated spatial regions (lower right). (E) Statistical analysis comparing the region of interest with random regions across 6 samples, *P < 0.05, **P < 0.01. (F to K) Spatial neighbor analysis based on Euclidean distance: using PROM1^+^SMAD5^+^ cells as reference points, distances from other spots (DCN^+^TM4SF1^+^ and ADIPOQ^+^NOTCH4^+^) to reference points were calculated. Top: Heatmap where color intensity represents distance; bottom: density curves of different cell types plotted against the above distances. (L) Spatial distribution of PROM1^+^SMAD5^+^Claudin1^+^ cells and PROM1^+^SMAD5^+^Claudin1^−^ cells across 6 samples. (M) Case 1, left: based on the differentiation timing of these 2 subpopulations, local trajectories were constructed via functional modeling to calculate the spatiotemporal distances between the PROM1^+^SMAD5^+^Claudin1^−^ subpopulation (with relatively stronger stemness, defined as 0) and the PROM1^+^SMAD5^+^Claudin1^+^ subpopulation (with relatively weaker stemness, defined as 1); middle: differentiation trees were used to illustrate the inter-subpopulation differentiation trajectories from PROM1^+^SMAD5^+^Claudin1^−^ to PROM1^+^SMAD5^+^Claudin1^+^ according to the differentiation sequence; right: transition genes along differentiation branches. Genes that increase or decrease along the trajectory from PROM1^+^SMAD5^+^Claudin1^−^ to PROM1^+^SMAD5^+^Claudin1^+^ are defined as transition genes, with up-regulated genes shown in purple and down-regulated genes in ginger yellow. Same with Case 2 (N) and Case 3 (O). Abbreviations: PROM1, prominin 1; SMAD5, SMAD family member 5; ADIPOQ, adiponectin; NOTCH4, notch receptor 4; DCN, decorin; TM4SF1, transmembrane 4 L six family member 1; H&E, hematoxylin and eosin staining.

We collected samples from 6 patients with HER2-positive breast cancer and performed spatial transcriptome studies using the 10x Genomics spatial platform. Based on RCTD results, we identified the 2 most abundant cell types in each spot (Fig. S8A and B). Subsequently, these 3 subpopulations were mapped onto histological H&E slides to determine their spatial distribution (Fig. 6A). Notably, *ADIPOQ^+^NOTCH4*^+^ cells were only detected in 3 out of 6 samples. Therefore, in the preceding spatial protein analysis, we focused on *PROM1^+^SMAD5^+^* and *DCN^+^TM4SF1*^+^ subpopulations—given their high occurrence probability. Finally, we assessed EGFR activation levels in these subpopulations and found significantly higher EGFR activation in *PROM1^+^SMAD5*^+^ cells compared with *PROM1^+^SMAD5^−^* cells (Fig. S8C).

To link these cell subpopulations with pathological phenotypes, we divided regions into random regions (RRs) and ROIs based on pathological malignancy. Statistical analysis of the subpopulation counts in each region showed that these 3 subpopulations (or *PROM1^+^SMAD5*^+^ and *DCN^+^TM4SF1*^+^ subpopulations in some cases) were mainly concentrated in invasive cancer areas (Fig. 6B to E and Fig. S8D to F). To further extend the relevance of these findings to more cancer types, similar enrichment patterns were observed in additional cases, including 4 breast cancer samples, 2 colorectal cancers, 2 gynecological cancers (ovarian cancer and cervical cancer), 2 prostate cancers (prostate cancer and prostate acinar cell carcinoma), 1 lung cancer (lung squamous cell carcinoma), and 1 brain cancer (glioblastoma) (Fig. S9). Eventually, we depicted these critical pathological areas through stylized abstract artwork (Fig. S10). These findings indicate that similar stromal–epithelial coexistence patterns are present across other cancer types, supporting the notion that this niche represents a shared pathological feature of aggressive tumors. Spatial neighbor analysis based on Euclidean distance metrics revealed that in all samples, both the *DCN^+^TM4SF1*^+^ and *ADIPOQ^+^NOTCH4*^+^ subpopulations (absent in some samples) were located in close proximity to *PROM1^+^SMAD5^+^* cells (Fig. 6F to K).

We previously confirmed that *PROM1^+^SMAD5^+^Claudin1*^−^ cells exhibit greater stemness than their *PROM1^+^SMAD5^+^Claudin1*^+^ counterparts (Fig. 3). In this study, we spatially analyzed regional evolutionary changes driven by stemness gradients. Using spatial coordinates of *PROM1^+^SMAD5^+^Claudin1*^−^ and *PROM1^+^SMAD5^+^Claudin1*^+^ subpopulations across 6 tissue samples (Fig. 6L), we built local differentiation trajectories via computational algorithms, quantifying spatiotemporal distances between subpopulations designated as state 0 (*PROM1^+^SMAD5^+^Claudin1*^−^) and state 1 (*PROM1^+^SMAD5^+^Claudin1*^+^). Taking Case 1 to 3 as example (Fig. 6M to O), differentiation trees visualized transition pathways from *PROM1^+^SMAD5^+^Claudin1*^−^ cells (derived from yellow spatial regions) to *PROM1^+^SMAD5^+^Claudin1*^+^ cells (from purple regions). We identified transition genes with monotonic expression shifts along this differentiation trajectory. Notably, alongside *CLDN1* (up-regulated as stemness declines), the down-regulated genes included *SERPINE1*, a key player in pEMT. This recurrence highlights the potential role of *SERPINE1* in regulating both pEMT and stemness.

Taken together, the 3 key cell subpopulations are predominantly enriched in invasive tumor regions across multiple cancer types, indicating a universal stromal–epithelial colocalization pattern in aggressive malignancies; meanwhile, Claudin1 and SERPINE1 are dynamically regulated during the stemness-related differentiation of PROM1^+^SMAD5^+^ cells, jointly modulating cell stemness and pEMT progression.

### Spatial regionalization characteristics of metabolites centered on the *PROM1^+^SMAD5*^+^ subpopulation

Prior analyses have elucidated on how these 3 cell subpopulations are related to pathological regions at the spatial transcriptome level. Investigating the metabolic variation patterns within these regions and their connections to the target genes is essential. To address this issue, we developed a method that merges spatial transcriptomics with spatial metabolomics (Fig. S11A). Next, we performed a step-by-step analysis of the pathways linked to *CYP3A4*. The tamoxifen metabolic pathway showed a substantial increase in the *CYP3A4*-derived metabolite N-desmethyltamoxifen (Fig. S11B).

**Fig. 7.**
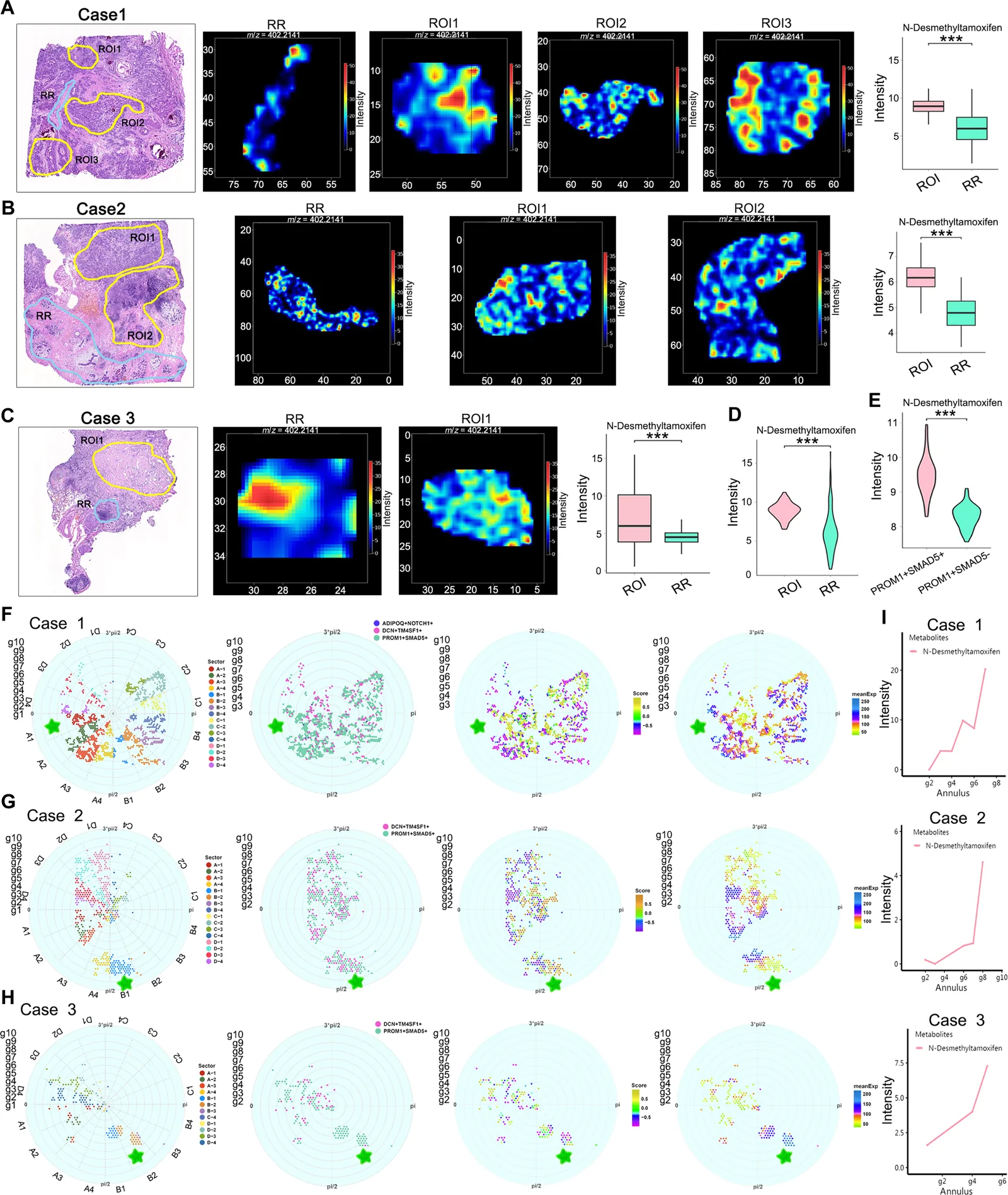
Spatial regionalization characteristics of metabolites centered on the PROM1^+^SMAD5^+^ subpopulation. Detailed division of the 3 samples (A to C) into point-to-point histopathological regions, with presentation of the divided N-desmethyltamoxifen ion density, and statistical analysis of the ion density in RR and ROIs. (D) Differential ion density analysis of N-desmethyltamoxifen between RR and ROIs of the 6 samples. ***P < 0.001. (E) Differential ion density analysis of the metabolite N-desmethyltamoxifen between the PROM1^+^SMAD5^+^ and PROM1^+^SMAD5^−^ cell subpopulations after the integration of 6 samples. ***P < 0.001. (F to H) First column: for Cases 1 to 3, tissue spatial regions were divided into 16 zones with the tissue center point as the center, and mapping diagrams of the 16 zones were plotted. Second column: SSCC clustering of the 3 subpopulations or 2 subpopulations, with clustering results mapped in the spatial dimension. Third column: Based on the activation of the EGFR pathway, intensity distribution maps were plotted in spatial radiation zones. Fourth column: based on the intensity of N-desmethyltamoxifen metabolites, intensity distribution maps were plotted in spatial radiation zones. Letters A to D represent quadrants. Centered on the central point, they divide the entire 2-dimensional plane into sixteen quadrants. The letter g stands for gradient; the numeral following g denotes distinct gradients radiating outward from the central point. These numbers extend equidistantly in all directions starting from the center (defined as 0). Numerical values such as 10, 9, 8, and so on correspond to scanning step positions, where higher values represent a greater distance away from the center. (I) The specific metabolite N-desmethyltamoxifen exhibited enriched and regular gradient changes in PROM1^+^SMAD5^+^ cell dense regions. Abbreviations: PROM1, prominin 1; SMAD5, SMAD family member 5; EGFR, epidermal growth factor receptor; RR, random region; ROI, region of interest; SSCC, spatial shrunken centroids clustering.

Point-to-point information analysis confirmed the enrichment of these 3 subpopulations in the ROIs, as defined by their spatial distribution. N-desmethyltamoxifen levels in these ROIs were significantly elevated compared with those in RRs (Fig. 7A to D and Fig. S11C to E). This metabolite also showed a consistent trend: its levels were notably higher in the *PROM1^+^SMAD5*^+^ population than in the *PROM1^+^SMAD5^−^* group (Fig. 7E).

Using the tissue centers as starting points, we analyzed the metabolic concentration gradients spreading outward across the 6 samples. Our analytical framework combined spatial metabolomics and transcriptomics, and adopted a randomized 6-point linear sampling strategy across 6 tissue sections. First, each tissue section was radially divided into 16 concentric zones from the center, and spatial mapping was performed for each zone (Fig. 7F to H, first column). Next, SSCC of *ADIPOQ^+^NOTCH4*^+^ and/or *DCN^+^TM4SF1*^+^ and *PROM1^+^SMAD5*^+^ cells were overlaid onto spatial coordinates to visualize their distribution patterns (Fig. 7F to H, second column). Subsequently, spatial distribution maps of EGFR pathway activity (Fig. 7F to H, third column) and tamoxifen metabolic pathway activity (Fig. 7F to H, fourth column) were created based on the intensity gradients across the radial zones. Finally, N-desmethyltamoxifen metabolites accumulated in a gradient-specific manner and were enriched in regions with high densities of *PROM1^+^SMAD5*^+^ cells (Fig. 7I). This suggests that these subpopulations work together to regulate local metabolic microenvironments. Notably, the radial interface (marked with pentagrams), which could not be identified by conventional pathological examination (confirmed by blinded analysis by 3 pathologists), exhibited unique N-desmethyltamoxifen metabolic characteristics. This highlights the distinctive capability of integrated spatial omics to detect micro-regional differences that traditional histology cannot, thus offering new perspectives for minimally invasive diagnosis and therapeutic targeting.

Notably, targeted metabolomic analysis further confirmed that N-desmethyltamoxifen was significantly enriched in *PROM1^+^SMAD5*^+^ cell subpopulations relative to *PROM1^+^SMAD5^−^* subpopulations upon tamoxifen treatment (Fig. S11F to H), directly linking elevated CYP3A4 activity in TICs to enhanced tamoxifen metabolism. Collectively, these findings establish N-desmethyltamoxifen as a robust functional readout of the EGFR–SMAD5–CYP3A4 axis in HER2-positive breast cancer, and a reliable surrogate for CYP3A4 enzymatic activity and TIC-driven malignant progression. Importantly, our focus on tamoxifen and its key metabolite does not support tamoxifen monotherapy for this tumor subtype—instead, it leverages tamoxifen’s well-characterized metabolic profile to decode the mechanistic link between TIC stemness, metabolic remodeling, and drug resistance, while validating N-desmethyltamoxifen as a potential metabolic marker for aggressive HER2-positive breast cancer.

In short, the combined spatial transcriptomics and metabolomics analysis proves that the EGFR–SMAD5–CYP3A4 axis drives the accumulation of N-desmethyltamoxifen in tumor focal regions, and this metabolite can act as a reliable metabolic biomarker to reflect the activity of oncogenic signaling and malignant progression of PROM1^+^SMAD5^+^ cells.

### Spatial in situ analysis of breast cancer metastasis and chemoresistance associated with *PROM1^+^SMAD5*^+^ subpopulations

The aforementioned in vivo experiments in mice had shown that these 3 cell subpopulations were enriched in the primary tumor site, metastatic lymph nodes and lungs. Similarly, such enrichment of subpopulations was also observed in the metastatic lymph nodes and lungs of breast cancer patients. Furthermore, in vitro experiments and spatial transcriptomics revealed that the PROM1^+^SMAD5^+^ subpopulation contained a PROM1^+^SMAD5^+^Claudin1^−^ subpopulation with increased stemness and metastatic potential. In patients with breast cancer, early metastatic events are typically characterized by lymph node involvement. Cancer cells from the primary breast tumor spread to the lymph nodes, leaving distinct pathological traces of embolic cancer cell clusters in the blood vessels [50]. Therefore, we investigated the enrichment of key subpopulations within these vascular cancer emboli to determine whether these cell subpopulations were critical for early metastasis of breast cancer. In addition, chemotherapy-induced resistance often resulted in the expansion of CSCs or the induction of more aggressive CSCs. Therefore, patients treated with chemotherapy were included in our experimental design.

We selected 21 pairs of HER2-positive breast cancer patients with lymph node metastasis, targeting typical embolic cancer cell clusters in the primary tumors and lymph node regions, and conducted spatial in situ sequencing using 10x Xenium. The cohort was structured to account for chemotherapy and subsequent pulmonary metastasis. In HER2-positive breast cancer patients without or with distant pulmonary metastasis, chemotherapy significantly increased the abundance of these 4 subpopulations within embolic cancer cell clusters in both primary tumors and lymph node metastatic sites (Fig. S12A and B). Among patients who did not or did receive chemotherapy, those with distant pulmonary metastasis showed significant enrichment of these 4 subpopulations in embolic clusters in both primary tumors and lymph node metastases (Fig. S12C and D).

**Fig. 8.**
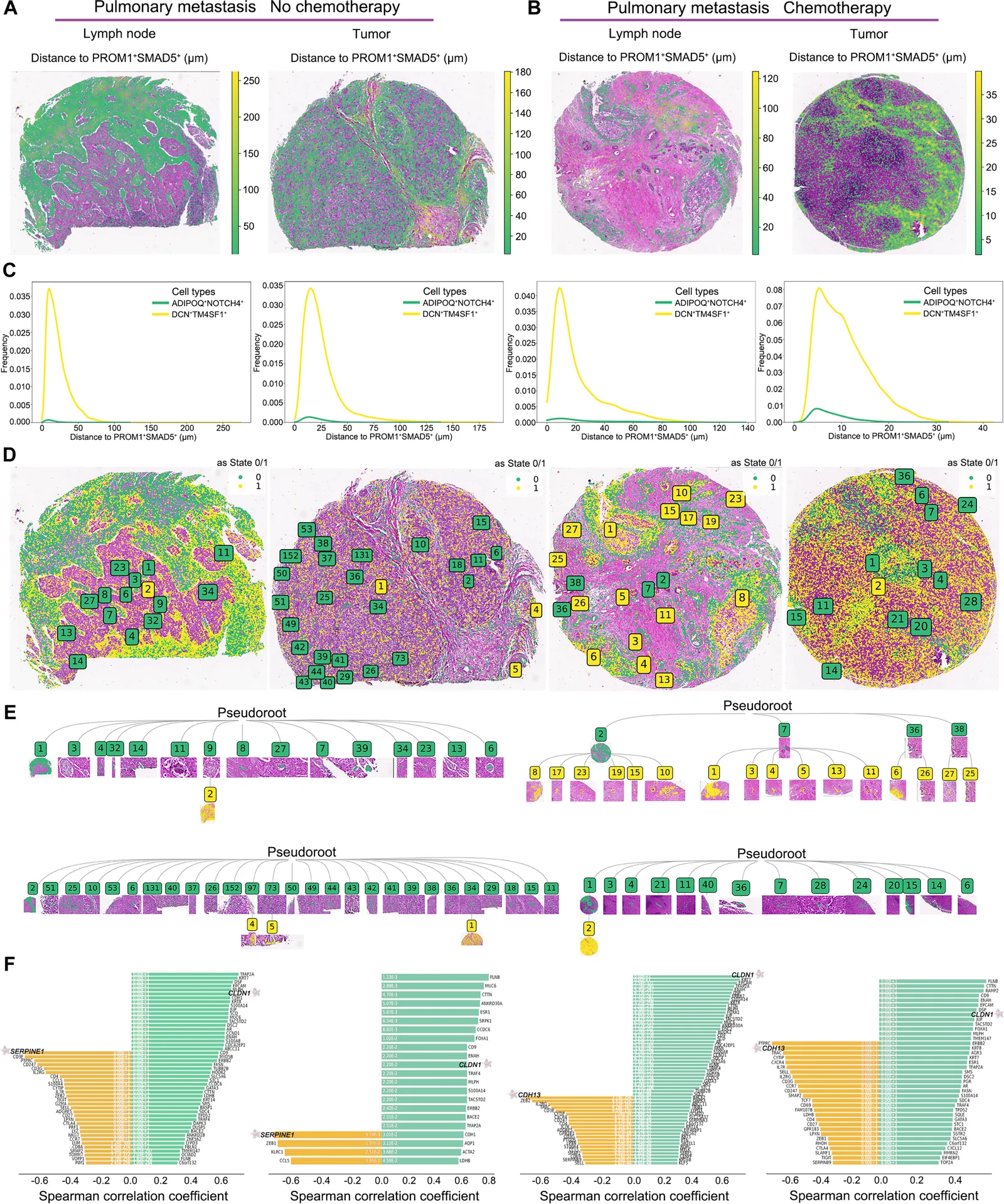
Spatial in situ analysis of breast tumor metastasis and chemoresistance associated with PROM1^+^SMAD5^+^ subpopulations. (A and B) Spatial in situ detection of embolic cancer cell clusters in lymph nodes and primary breast tumors from HER2-positive breast cancer patients with distant pulmonary metastasis, stratified by chemotherapy-naive (A) and chemotherapy-treated status (B). Euclidean distance-based spatial neighbor analysis: PROM1^+^SMAD5^+^ cells were used as reference points to measure the distances from other cell populations (DCN^+^TM4SF1^+^ and ADIPOQ^+^NOTCH4^+^) to these reference points. Heatmap with color intensity indicating distance. (C) Density curves of various cell types in matched samples, plotted against the aforementioned distance. (D) In the matched samples, local trajectories were built through functional modeling based on the differentiation timing of these 2 subpopulations, aiming to compute the spatiotemporal distances between the PROM1^+^SMAD5^+^Claudin1^−^ subpopulation (with relatively higher stemness, designated as 0) and the PROM1^+^SMAD5^+^Claudin1^+^ subpopulation (with relatively lower stemness, designated as 1). (E) In the matched samples, differentiation trees were employed to demonstrate the inter-subpopulation differentiation paths from PROM1^+^SMAD5^+^Claudin1^−^ to PROM1^+^SMAD5^+^Claudin1^+^ according to the differentiation order. (F) In the matched samples, transition genes across differentiation branches: Genes that rise or fall along the trajectory from PROM1^+^SMAD5^+^Claudin1^−^ to PROM1^+^SMAD5^+^Claudin1^+^ are defined as transition genes, with up-regulated genes displayed in green and down-regulated genes in ginger yellow. Abbreviations: HER2, human epidermal growth factor receptor 2; PROM1, prominin 1; SMAD5, SMAD family member 5; ADIPOQ, adiponectin; NOTCH4, notch receptor 4; DCN, decorin; TM4SF1, transmembrane 4 L six family member 1.

Next, we focused on breast cancer patients with pulmonary metastasis, including those receiving or not receiving chemotherapy, by analyzing the distribution of the 3 subpopulations within the dense embolic niches of primary breast tumors and lymph nodes. Spatial neighbor analysis based on Euclidean distance metrics showed that both DCN^+^TM4SF1^+^ and ADIPOQ^+^NOTCH4^+^ cells are located close to PROM1^+^SMAD5^+^ cells (Fig. 8A to C).

Using the spatial coordinates of the PROM1^+^SMAD5^+^Claudin1^−^ and PROM1^+^SMAD5^+^Claudin1^+^ subpopulations in these embolic samples, we employed computational algorithms to construct local differentiation trajectories, quantifying the spatiotemporal distances between subpopulations designated as state 0 (PROM1^+^SMAD5^+^Claudin1^−^) and state 1 (PROM1^+^SMAD5^+^Claudin1^+^) (Fig. 8D). Differentiation trees were generated to visualize transition pathways from PROM1^+^SMAD5^+^Claudin1^−^ cells (derived from teal-colored spatial regions) to PROM1^+^SMAD5^+^Claudin1^+^ cells (from ginger-colored regions) (Fig. 8E). We also identified transition genes with monotonic expression shifts along the differentiation trajectory (Fig. 8F). Notably, along with the up-regulation of Claudin1 (consistent with decreasing stemness during differentiation), the down-regulated genes included not only *SERPINE1* (closely linked to pEMT) but also *CDH13*. This suggests that these genes play a role in the regulation of both pEMT and stemness.

Ultimately, chemotherapy and distant pulmonary metastasis induce the enrichment of PROM1^+^SMAD5^+^ cells and related stromal subpopulations within vascular cancer emboli during early metastasis; Claudin1, SERPINE1, and CDH13 are dynamically expressed during the in situ differentiation of PROM1^+^SMAD5^+^ cells, linking cell stemness, pEMT, chemoresistance, and early metastatic events in HER2-positive breast cancer.

## Discussion

We identified a PROM1^+^SMAD5^+^ tumor-initiating subpopulation with pEMT characteristics in HER2-positive breast cancer and delineated its “affinity niche” interactions with ADIPOQ^+^NOTCH4^+^ adipocytes and DCN^+^TM4SF1^+^ fibroblasts. This tripartite network drives metastasis and chemoresistance through 2 key mechanisms: activation of the EGFR–SMAD5–CYP3A4 axis via the COPA–EGFR ligand–receptor interaction, and down-regulation of *Claudin1* in PROM1^+^SMAD5^+^ cells by exosomal miR-671-3p secreted from stromal cells (Fig. S13). Clinically, the enrichment of these 3 subpopulations relates with high malignancy, lymph node/pulmonary metastasis, and poor prognosis. Spatial multi-omics further confirmed their specific aggregation in invasive tumor regions, necrotic areas, and metastatic foci, offering new insights into breast cancer progression.

The pEMT phenotype of PROM1^+^SMAD5^+^ cells, coupled with their positioning at the initiation point of pseudotime transitions, explains their high plasticity as metastatic “seeds” [4]. Stromal cells regulate this subpopulation through coordinated mechanisms: COPA–EGFR-mediated signaling enhances stemness and drug resistance, whereas exosomal miR-671-3p-induced Claudin1 down-regulation disrupts tight junctions to promote invasiveness. This coordinated regulation transcends the traditional focus on individual stromal cell types, highlighting the “trinity niche” as a functional unit critical for tumor progression.

The enrichment of PROM1^+^SMAD5^+^ cells is a potential prognostic marker of poor outcomes in HER2-positive breast cancer. Their spatial colocalization with stromal cells in vascular-dense and necrotic regions guides targeted biopsy strategies. Interventions targeting the COPA–EGFR axis (e.g., combined EGFR inhibitors and COPA antibodies) or blocking miR-671-3p transfer may suppress metastasis and reverse drug resistance, particularly in patients with lymph node metastasis or post-chemotherapy recurrence.

Our findings advance the understanding of TICs in breast cancer by identifying a PROM1^+^SMAD5^+^ subpopulation, which fills a gap in the current knowledge of hybrid E/M states. Unlike traditional CSC markers such as CD44^+^CD24^−^ (mesenchymal) [9] and ALDH1A3^+^ (epithelial) [10] that represent extreme ends of the EMT spectrum, PROM1^+^SMAD5^+^ cells exhibit a pEMT phenotype, aligning with emerging evidence that hybrid E/M states are pivotal for metastatic competence. This echoes studies on K14^+^E-cadherin^+^ subpopulations driving collective invasion in other cancers [13], and extends to HER2-positive breast cancer by linking such cells to specific stromal crosstalk.

In terms of stromal interactions, previous research has individually implicated adipocytes and fibroblasts in breast cancer progression: adipocytes via cytokine secretion [14], and fibroblasts through ECM remodeling [15,16]. Our work demonstrates the coordinated role of ADIPOQ^+^NOTCH4^+^ and DCN^+^TM4SF1^+^ cells in parallelly regulating TIC behavior, a dynamic rarely explored in breast cancer.

Regarding exosomal signaling, while exosomes mediate intercellular communication in cancer, our identification of miR-671-3p as a key regulator of *Claudin1* adds specificity to this field. This builds on studies linking Claudin family dysregulation to metastasis [51] and provides a concrete mechanism for such effects in breast cancer.

Our integrated spatial multi-omics analysis revealed markedly elevated CYP3A4-mediated N-desmethyltamoxifen production in PROM1^+^SMAD5^+^ TICs of HER2-positive breast cancer, and functional assays confirmed that this metabolite is significantly enriched in PROM1^+^SMAD5^+^ relative to PROM1^+^SMAD5^−^ subpopulations upon tamoxifen treatment, linking elevated CYP3A4 activity in TICs to enhanced tamoxifen metabolism and endocrine resistance. While tamoxifen is not a standard therapy for HER2-positive breast cancer, its metabolite N-desmethyltamoxifen serves as a robust functional readout of the EGFR–SMAD5–CYP3A4 axis that drives TIC stemness and drug resistance, and its enrichment in metastatic foci and post-chemotherapy samples validates its potential as a metabolic marker for aggressive HER2-positive breast cancer, which may guide patient stratification and the development of combination therapies targeting this key axis. Finally, our findings on *CYP3A4* up-regulation via the EGFR–SMAD5 axis expand on prior reports of the role of CYP3A4 in drug metabolism and resistance [52], by situating it within a stroma-regulated pathway specific to PROM1^+^SMAD5^+^ cells, thus explaining why HER2-positive tumors often develop resistance to therapy.

Our study also had some limitations. The use of mouse models (e.g., PY8119 cell xenografts) may not fully recapitulate human TME complexity, and larger cohorts are needed to validate the parabiosis-derived migration patterns of the 3 subpopulations. In addition, the direct regulatory link between N-desmethyltamoxifen and *CYP3A4* in spatial metabolomics requires further validation.

## Conclusions

This study uncovers a stroma-regulated mechanism driving metastasis and drug resistance in HER2-positive breast cancer, centered on the PROM1^+^SMAD5^+^ subpopulation and its “trinity niche”. These findings provide a basis for precision therapies targeting tumor–stroma interactions, with the potential to improve outcomes in patients with aggressive HER2-positive breast cancer.

## Ethical Approval

Animal experiments were approved by the Institutional Animal Care and Use Committee (No. 2023AWS082) and human studies were approved by the Shanghai General Hospital Medical Ethics Committee (No. 2021SQ203), with informed consent from all patients.

## Data Availability

Processed data are deposited in the National Genomics Data Center (OMIX006245).
