# Supplementary material for: The PROM1+SMAD5+ Tumor-Initiating Subpopulation Shapes Premetastatic Niches through Spatial Multi-Omics Landscapes in HER2-Positive Breast Cancer
Source: Cancer Commun (Lond). 2026 Sep 9;46:0046. doi: 10.34133/cancomm.0046 (PMC13554431; doi:10.34133/cancomm.0046)
Supplement: Supplementary file 1 [file cancomm.0046.f1.docx]

**Supplementary Figures**

**Supplementary Figure S1**. **GO enrichment and pseudotime analysis revealing EMT-related features in SMAD5-high epithelial cell subpopulations.** (A) GSVA GO showed the significantly enriched biological functions of each of the 11 epithelial cells subsets. Pseudotime analysis of gene expression variation in high-plasticity genes. (B) Heatmap showing transition modules. These modules capture key gene sets during the pseudotime branch. They correspond to the transition toward cell states 1 and 3. (C) Dot plots showing GO enrichment results. These results are specific to module 2.

Abbreviations: GO, Gene Ontology; EMT, Epithelial-Mesenchymal Transition;

**Supplementary Figure S2. Enrichment frequency and stemness/tumorigenic capacity validation of PROM1^+^SMAD5^+^ epithelial subpopulations.** (A) Line graph showing percentage differences of PROM1⁺SMAD5⁺ subpopulations across 11 epithelial cell subpopulations. (B) Representative images of mammospheres. (C) Mammosphere formation differences between PROM1⁺SMAD5⁺ cells and PROM1⁺SMAD5⁻ cells at the cell numbers of 1, 10, and 50, **P* < 0.05. (D−E) Comparison and statistics of tumorigenic differences between PROM1⁺SMAD5⁺ cells and PROM1⁺SMAD5⁻ cells in immunodeficient mice under the conditions of 1,000, 100, and 10 cells. (F−G) The tumors formed by 1,000 first-generation PROM1⁺SMAD5⁺ cells and PROM1⁺SMAD5⁻ cells were re-sorted to obtain PROM1⁺SMAD5⁺ cells and PROM1⁺SMAD5⁻ cells respectively, and then 1,000 cells of each were inoculated to compare and statistically analyze the tumorigenic differences. (H−I) The tumors formed by 1,000 second-generation PROM1⁺SMAD5⁺ cells and PROM1⁺SMAD5⁻ cells were re-sorted to obtain PROM1⁺SMAD5⁺ cells and PROM1⁺SMAD5⁻ cells respectively, and then 1,000 cells of each were inoculated to compare and statistically analyze the tumorigenic differences.

Abbreviations: PROM1, Prominin 1; SMAD5, SMAD Family Member 5.

**Supplementary Figure S3 Spatial proximity and interregional interaction analysis between EPCAM^+^HER2^+^ cells and PROM1^+^SMAD5^+^ cells in selected tumor regions of interest.** In regions of interest (ROI) 1 (A), ROI 4 (D-E), ROI 5 (H), ROI6 (K), the nearest neighbor distance between each EPCAM^+^HER2^+^ cell and its closest PROM1^+^SMAD5^+^ cell was calculated. (B, F, I, L) Corresponding region interaction network diagrams, where the size of the nodes indicates the number of elements (cells) within the neighborhood, and the straight lines represent shared boundaries between neighborhoods. The line thickness corresponds to the percentage of shared border, with thicker lines indicating a higher proportion of shared boundaries. (C, G, J, M) Region interaction heatmaps showing specific values for the interactions between different regions.

Abbreviations: ROI, Regions of Interest; EPCAM, Epithelial Cell Adhesion Molecule; HER2, Human Epidermal Growth Factor Receptor 2.

**Supplementary Figure S4 Clinicopathological correlation of PROM1^+^SMAD5^+^ cells and external single-cell dataset validation of epithelial subpopulations and SMAD5/PROM1 expression.** Correlation analysis of PROM1^+^SMAD5^+^ cell proportion with breast cancer clinicopathological characteristics, including molecular subtypes (A), histological grade (B), **P* < 0.05. Validation of epithelial cell subsets and SMAD5/PROM1 expression patterns in independent breast cancer single-cell datasets. (C) Unsupervised clustering combined with UMAP visualization reveals 8 distinct cell types in the GSE161529 dataset. (D) UMAP plot showing epithelial cells, which were defined using markers from external data and further delineated into 5 distinct subsets. (E) UMAP visualization illustrating the mapping results between the 5 epithelial subclusters identified in this dataset and the 3 epithelial subsets reported in our study. (F) Sankey diagram demonstrating the correlation between the 5 literature-defined epithelial subclusters and their corresponding 3 subsets identified in our study. (G) UMAP projection showing the expression patterns of SMAD5 and PROM1 across all epithelial subclusters. (H) Unsupervised clustering and UMAP visualization exhibit nine distinct cell types in the GSE176078 dataset. (I) UMAP plot presenting epithelial cells, which were defined by markers from third-party literature and subdivided into 9 distinct subsets. (J) UMAP visualization displaying the matching mapping results between the 9 epithelial subclusters in this dataset and the 6 epithelial subsets described in our study. (K) UMAP projection depicting the expression of SMAD5 and PROM1 in each epithelial subcluster. (L) Dot plot showing the expression intensity of SMAD5 and PROM1 genes in HER2-positive and HER2-negative breast cancer cells. (M) Violin plot quantifying the percentage of PROM1^+^SMAD^+^ cells in HER2-positive versus HER2-negative breast cancer samples.

Abbreviations: PROM1, Prominin 1; SMAD5, SMAD Family Member 5; UMAP, Uniform Manifold Approximation and Projection; HER2, Human Epidermal Growth Factor Receptor 2.

**Supplementary Figure S5 Validation of COPA-EGFR binding via SPR and immunofluorescence imaging in defined stromal subsets and PROM1^+^SMAD5^+^ cells.** (A) Schematic diagram of Surface Plasmon Resonance (SPR). (B) Kinetic curves from subsequent concentration-dependent SPR analysis showing the binding of COPA to EGFR. (C) Schematic diagram of the experimental design steps. (D) COPA was detected after concentration from the supernatant of stromal cells as shown in the figure. Panoramic view (E) of the interaction between COPA and EGFR in PROM1^+^SMAD5^+^ cells detected from the supernatants of DCN^+^TM4SF1^+^ and DCN^+^TM4SF1^-^, and a magnified close-up view (F) of one of the cells. Panoramic view (G) of the interaction between COPA and EGFR in PROM1^+^SMAD5^+^ cells detected from the supernatants of ADIPOQ^+^NOTCH4^+^ and ADIPOQ^+^NOTCH4^-^, and a magnified close-up view (H) of one of the cells.

Abbreviations: SPR, Surface Plasmon Resonance; COPA, Coatomer Protein Complex Subunit Alpha; EGFR, Epidermal Growth Factor Receptor; PROM1, Prominin 1; SMAD5, SMAD Family Member 5; DCN, Decorin; TM4SF1, Transmembrane 4 L Six Family Member 1; ADIPOQ, Adiponectin; NOTCH4, Notch Receptor 4; CYP3A4, Cytochrome P450 Family 3 Subfamily A Member 4; qRT-PCR, quantitative Real-Time Polymerase Chain Reaction

**Supplementary Figure S6 Drug sensitivity and molecular phenotypes of PROM1^+^SMAD5^+^ cells.** (A) PROM1⁺SMAD5⁺ cells were treated with 1µmol/L Erlotinib for 4 and 48 h or left untreated, and the cell counting kit-8 (CCK8) assay was used to detect the OD450 value as an indicator of cell viability. Flow cytometry was used to detect the double-positive percentage (B) and quantitative statistics (C) of PROM1⁺SMAD5⁺ cells at 0, 24, and 48 h. (D-E) Quantitative analysis corresponding to Figure 2F. **P* < 0.05, ***P* < 0.01. (F) Western blot analysis showing changes in CYP3A4 protein levels in the PROM1^+^SMAD5^+^ subpopulation following *SMAD5* knockdown. (G) Quantitative analysis of panel F. ***P* < 0.01. (H-I) Quantitative analysis corresponding to Figure 2G. ***P* < 0.01. (J) The IC50 of taxol in PROM1⁺SMAD5⁺ cells from indicated monoculture and coculture systems. Cell viability was assessed by CCK8 assay based on OD450 values after 48h treatment. **P* < 0.05. (K) Apoptosis analysis of PROM1^+^SMAD5^+^ (treated with 10 µmol/L taxol for 48 h) by Annexin V/PI staining in the indicated monoculture and coculture systems.

Abbreviations: CCK8, Cell Counting Kit-8; OD450, Optical Density at 450 nm; PROM1, Prominin 1; SMAD5, SMAD Family Member 5; IC50, Half Maximal Inhibitory Concentration; PI, Propidium Iodide; CYP3A4

Cytochrome P450 3A4.

**Supplementary Figure S7 Exosomal miR-671-3p regulates Claudin-1 and phenotypes of PROM1^+^SMAD5^+^ cells.** (A) Electron microscopy of patient-derived exosomes. Western blot analysis (B) and quantitative statistics (C-D) of exosome markers: CD63 (exosomal marker) and MMP9 (ectosome marker). (E) Volcano plot highlighting significantly differentially expressed miRNAs (upregulated and downregulated) between breast cancer and adjacent normal tissue groups. (F) Combined workflow diagram of single-cell nuclear transcriptome and exosome small RNA sequencing from matched tissue samples. (G) ssGSEA heatmap depicting enrichment of exosomal pathways across eight cell populations. (H) Quantitative analysis of panel 3I**,** **P* < 0.05. (I) Schematic diagram of the experimental design for the binding of miR-671-3p to the 3'UTR of Claudin 1 mRNA. (J) The effect of mimic miR-671-3p and anti-miR-671-3p constructs on the luciferase activity of the 3′-UTR binding of the wild type or mutant Claudin 1, respectively; the effect of mimic miR-671-3p and anti-miR-671-3p on PROM1^+^SMAD5^+^ cells was assessed using a luciferase reporter gene activity assay. (K) Schematic diagram of the experimental design for inhibiting exosome secretion. (L-M) PROM1^+^SMAD5^+^ cells were co-cultured with DCN^+^TM4SF1^+^ or ADIPOQ^+^NOTCH4^+^ cells, which were pre-transfected with GW4869, respectively. Western Blot (WB) analysis was used to examine the effects of GW4869 on Claudin 1 expression in PROM1^+^SMAD5^+^ cells. **P* < 0.05. (N) Schematic diagram of the experimental design for exosome-specific loss-of-function assay. (O) Quantitative Real-Time PCR (qPCR) analysis was used to examine the effect of extracellular vehicles (EVs) derived from DCN^+^TM4SF1^+^ or ADIPOQ^+^NOTCH4^+^ cells, which were pre-transfected with anti-miR-671-3p or anti-Ctrl constructs, on the expression of Claudin 1 mRNA in PROM1^+^SMAD5^+^ cells. **P* < 0.05.

Abbreviations: WB, Western Blot; CD63, Cluster of Differentiation 63; MMP9, Matrix Metalloproteinase 9; ssGSEA, single-sample Gene Set Enrichment Analysis; UTR, Untranslated Region; PROM1, Prominin 1; SMAD5, SMAD Family Member 5; DCN, Decorin; TM4SF1, Transmembrane 4 L Six Family Member 1; ADIPOQ, Adiponectin; NOTCH4, Notch Receptor 4.

**Supplementary Figure S8 Spatial distribution and EGFR signaling characteristics of PROM1^+^SMAD5^+^ cell subpopulations.** (A-B) Top two cell types with the highest proportion in each spatial spot, as determined by RCTD analysis. (C) Left: Feature plot of the gene module for EGFR signaling pathway, depicting spatial distribution patterns of the three subpopulations across six tissue samples. Right: Boxplot comparing EGFR signaling pathway activity between PROM1^+^SMAD5^+^ and PROM1^+^SMAD5^-^ subgroups, ***P* < 0.01. (D-F) Spatial localization of the three (or two) subpopulations overlaid on HE staining (Left); Pathological features of the delineated spatial regions (Right upper); Quantification of relative subpopulation percentages within the delineated spatial regions (Right low).

Abbreviations: EGFR, Epidermal Growth Factor Receptor; PROM1, Prominin 1; SMAD5, SMAD Family Member 5; DCN, Decorin;

**Supplementary Figure S9 Spatial distribution and quantitative quantification of three cell subsets across multiple cancers.** Spatial transcriptomes showing the coexistence of three subsets PROM1^+^SMAD5^+^, ADIPOQ^+^NOTCH4^+^, and DCN^+^TM4SF1^+^ cells among various cancer types. (A-D) Breast cancer: A: Invasive lobular carcinoma; B: Invasive ductal carcinoma with *in situ* component; C: Ductal carcinoma *in situ* with invasive cancer; D: Invasive ductal carcinoma. (E-F) Colorectal cancers: E: Colon cancer; F: Colorectal invasive adenocarcinoma. (G-H) Gynecological cancers: G: Ovarian serous papillary carcinoma; H: Cervical squamous cell carcinoma. (I-J) Prostate cancers: I: Prostatic acinar cell carcinoma; J: Invasive adenocarcinoma. (K) Lung squamous cell carcinoma. (L) Multiform glioblastoma. (M-R): Quantitative analysis of targeted subpopulations in the above-mentioned cancer types.

Abbreviations: PROM1, Prominin 1; SMAD5, SMAD Family Member 5; DCN, Decorin; TM4SF1, Transmembrane 4 L Six Family Member 1; ADIPOQ, Adiponectin; NOTCH4, Notch Receptor 4.

**Supplementary Figure S10 Ink-style schematic overview of three cell subset cohabitation across various cancers.** Detailed sketches showing cohabitation zones of three subsets PROM1^+^SMAD5^+^, ADIPOQ^+^NOTCH4^+^, and DCN^+^TM4SF1^+^ cells within various cancer types, illustrated with oriental ink painting techniques. (A-D) Breast cancer: A: Invasive lobular carcinoma; B: Invasive ductal carcinoma with *in situ* component; C: Ductal carcinoma *in situ* with invasive cancer; D: Invasive ductal carcinoma. (E-F) Colorectal cancers: E: Colon cancer; F: Colorectal invasive adenocarcinoma. (G-H) Gynecological cancers: G: Ovarian serous papillary carcinoma; H: Cervical squamous cell carcinoma. (I-J) Prostate cancers: I: Prostatic acinar cell carcinoma; J: Invasive adenocarcinoma. (K) Lung squamous cell carcinoma. (L) Multiform glioblastoma.

Abbreviations: PROM1, Prominin 1; SMAD5, SMAD Family Member 5; DCN, Decorin; TM4SF1, Transmembrane 4 L Six Family Member 1; ADIPOQ, Adiponectin; NOTCH4, Notch Receptor 4.

**Supplementary Figure S11 Multi-omics integration strategy for spatial transcriptome-metabolome and quantification of CYP3A4-mediated N-desmethyltamoxifen levels in distinct cell subsets.** (A) Flowchart of the joint analysis method for spatial transcriptomics and spatial metabolomics. (B) Left: Tamoxifen drug metabolism pathways. Right: Key molecules related to *CYP3A4*, with chemical structure of N-Desmethyltamoxifen showed. Detailed division of the 3 samples (C-E) into point-to-point histopathological regions, with presentation of the divided N-Desmethyltamoxifen ion density, and statistical analysis of the ion density in RR and ROIs (F, I, L), ****P* < 0.001. (F) Experimental workflow for the quantitative detection of metabolite N-desmethyltamoxifen. (G) Chromatography-mass spectrometry analysis of N-desmethyltamoxifen content in PROM1^+^SMAD5^+^ and PROM1^+^SMAD5^-^ subgroups. (H) Statistical analysis of the difference in N-desmethyltamoxifen content between PROM1^+^SMAD5^+^ and PROM1^+^SMAD5^-^ subgroups, ***P* < 0.01.

Abbreviations: CYP3A4, Cytochrome P450 Family 3 Subfamily A Member 4; RR, Random Region; ROI, Region of Interest; PROM1, Prominin 1; SMAD5, SMAD Family Member 5.

**Supplementary Figure S12 Cellular subset distribution in cancer embolic niches stratified by metastasis and chemotherapy in HER2-positive breast cancer.** Violin plot display the expression of the DCN^+^TM4SF1^+^, ADIPOQ^+^NOTCH4^+^, PROM1^+^SMAD5^+^, PROM1^+^SMAD5^+^Claudin1^-^ subpopulation in the cancer embolic niches of HER2-positve breast cancer patients, without (A) or with (B) distant lung metastasis, without (C) or with (D) chemotherapy, in the primary breast tumor and lymph node regions. **P* < 0.05, ***P* < 0.01, ****P* < 0.001, *****P* < 0.0001.

Abbreviations: PROM1, Prominin 1; SMAD5, SMAD Family Member 5; DCN, Decorin; TM4SF1, Transmembrane 4 L Six Family Member 1; ADIPOQ, Adiponectin; NOTCH4, Notch Receptor 4; LN, Lymph Node.

**Supplementary Figure S13 Abstract representation of the key research significance conveyed in the article.** Left panel: Cheongsam, a traditional Chinese garment, is used to represent Chinese women. Folding fans partially covering the face symbolize the unresolved research challenges in breast cancer. Based on the color shades of the cheongsam and the hand-drawn distribution of breast cancer cells in three major organs (breast, lymph nodes, and lungs), this part implies the phylogenetic theory of multi-organ metastasis of cancer. Right-peripheral area: It illustrates the combined application of various omics technologies to establish a systematic approach for addressing key issues.

Right-center area: It presents the key molecular mechanisms involved in the study.

**Supplementary Table S1 Antibodies**

| Reagent | Source | Identifier | Dilution |
| --- | --- | --- | --- |
| **Antibodies-TSA (Human)** | | |  |
| Anti-CD133 antibody [EPR20980-104] | Abcam | ab216323 | 1:500 |
| Anti-SMAD5 antibody [EP619Y] | Abcam | ab40771 | 1:50 |
| Anti-Adiponectin antibody [19F1] | Abcam | ab22554 | 1:100 |
| Anti-NOTCH4 antibody | Sigma-Aldrich | N5163 | 1:100 |
| **Antibodies-TSA (Mouse)** |  |  |  |
| CD133 (Prominin-1) Monoclonal Antibody (13A4), eBioscience™ | Invitrogen | 14-1331-82 | 1:50 |
| Anti-SMAD5 antibody [EP619Y] | Abcam | ab40771 | 1:50 |
| Anti-Decorin antibody [EPR24097-105] | Abcam | ab277636 | 1:1000 |
| TM4SF1 Polyclonal Antibody | Invitrogen | PA5-21119 | 1:200 |
| **Antibodies-Flow cytometry** |  |  |  |
| Panel 1 |  |  |  |
| Anti-Adiponectin antibody [19F1] | Abcam | ab22554 | 1:50 |
| Anti-NOTCH4 antibody | Sigma-Aldrich | N5163 | 1:50 |
| F(ab')2-Goat anti-Mouse IgG (H+L) Secondary Antibody, Qdot™ 705 | Invitrogen | Q11062MP | 1:200 |
| F(ab')2-Goat anti-Rabbit IgG (H+L) Cross-Adsorbed Secondary Antibody, APC | Invitrogen | 31984 | 1:200 |
| Panel 2 |  |  |  |
| Decorin (G-1) | SANTA CRUZ | sc-518096 | 1:50 |
| Anti-TM4SF1 antibody | Atlas Antibodies | HPA002823 | 1:50 |
| Goat anti-Mouse IgG (H+L) Highly Cross-Adsorbed Secondary Antibody, Alexa Fluor™ 790 | Invitrogen | A11357 | 1:200 |
| F(ab')2-Goat anti-Rabbit IgG (H+L) Cross-Adsorbed Secondary Antibody, APC | Invitrogen | 31984 | 1:200 |
| Panel 3 |  |  |  |
| CD133 (Prominin-1) Monoclonal Antibody (TMP4), PE, eBioscience™ | Invitrogen | 12-1338-42 | 1:100 |
| Anti-SMAD5 antibody [EP619Y] | Abcam | ab40771 | 1:100 |
| F(ab')2-Goat anti-Rabbit IgG (H+L) Cross-Adsorbed Secondary Antibody, APC | Invitrogen | 31984 | 1:200 |
| Panel 4 |  |  |  |
| CD133 (Prominin-1) Monoclonal Antibody (13A4), eBioscience™ | Invitrogen | 14-1331-82 | 1:100 |
| Anti-SMAD5 antibody [EP619Y] | Abcam | ab40771 | 1:100 |
| Donkey anti-Rat IgG (H+L) Highly Cross-Adsorbed Secondary Antibody, Alexa Fluor™ Plus 405 | Invitrogen | A48268 | 1:200 |
| F(ab')2-Goat anti-Rabbit IgG (H+L) Cross-Adsorbed Secondary Antibody, APC | Invitrogen | 31984 | 1:200 |
| Panel 5 |  |  |  |
| APC Anti-GFP antibody [EPR14104] | Abcam | ab283597 | 1:500 |
| **Antibodies-Confocol** |  |  |  |
| Anti-Adiponectin antibody [19F1] | Abcam | ab22554 | 1:50 |
| Anti-NOTCH4 antibody | Sigma-Aldrich | N5163 | 1:50 |
| Donkey anti-Mouse IgG (H+L) Highly Cross-Adsorbed Secondary Antibody, Alexa Fluor™ 488 | Invitrogen | A-21202 | 1:1000 |
| Donkey anti-Rabbit IgG (H+L) Highly Cross-Adsorbed Secondary Antibody, Alexa Fluor™ 555 | Invitrogen | A-31572 | 1:1000 |
| Decorin (G-1) | SANTA CRUZ | sc-518096 | 1:100 |
| Anti-TM4SF1 antibody | Atlas Antibodies | HPA002823 | 1:100 |
| Donkey anti-Goat IgG (H+L) Highly Cross-Adsorbed Secondary Antibody, Alexa Fluor™ Plus 647 | Invitrogen | A32849 | 1:1000 |
| Donkey anti-Mouse IgG (H+L) Highly Cross-Adsorbed Secondary Antibody, Alexa Fluor™ Plus 647 | Invitrogen | A32787 | 1:1000 |
| GFP Polyclonal Antibody | Invitrogen | PA5-143569 | 1:200 |
| Anti-SMAD5 antibody [EP619Y] | Abcam | ab40771 | 1:100 |

**Supplementary Table S2 Other Materials, Instruments and Software**

| **Critical Commercial Assays** |  |  |
| --- | --- | --- |
| Vectastain Elite ABC Kit (Standard) | VECTOR | PK-6100 |
| Opal Polaris 7color Manual IHC Kit | AKOYA | NEL8600KT |
| Tumor Dissociation Kit, human | Miltenyi Biotec | 130-095-929 |
| Tumor Dissociation Kit, mouse | Miltenyi Biotec | 130-096-730 |
| Dead Cell Removal Kit | Miltenyi Biotec | 130-090-101 |
| Duolink® In Situ Red Starter Kit Mouse/Rabbit | Sigma-Aldrich | #DUO92105 |
| CCK-8 | Beyotime, China | C0038 |
| Annexin V/PI Apoptosis Detection Kit | Molecular Probes | V13242 |
| Lipofectamine® RNAiMAX | Invitrogen, USA | 13778075 |
| Rat tail collagen | Corning, NY, USA | 354236 |
| Vazyme Dual-Luciferase Reporter Assay Kit | Vazyme Biotech | Cat# DL101-01 |
| **Chemicals** |  |  |
| 10% neutral formalin | Solarbio | G2161 |
| ProLong Diamond antifade mountant | Life | P36965 |
| MACS Tissue Storage Solution, 100mL | Miltenyi Biotec | 130-100-008 |
| Red Blood Cell Lysis Buffer | Solarbio | R1010 |
| BD FACSDiva™ CS&T research beads (CS&T research beads) | BD Biosciences | 655050 |
| Anti-Mouse Ig, κ/Negative Control (BSA) Compensation Plus (7.5 µm) Particles Set | BD Biosciences | 560497 |
| UltraComp eBeads™ Compensation Beads | Thermo Fisher | 01-2222-42 |
| Flow Cytometry Staining Buffer | Thermo Fisher | 00-4222-57 |
| Fixation Buffer | BioLegend | 420801 |
| Intracellular Staining Perm Wash Buffer (10X) | BioLegend | 421002 |
| Antibody buffer | Keygentec, China | KGF005 |
| SRP Experiments |  |  |
| CM5 Sensor Chip | Cytiva | BR100530 |
| Amine Coupling Kit | Cytiva | BR100050, BR100349 |
| Sodium Acetate Buffer (pH 4.0/4.5/5.0) | Cytiva | BR100350, BR100351 |
| SRM/MRM Experiments |  |  |
| Formic Acid (FA) | Thermo Fisher | 6450 |
| Acetonitrile (ACN) | Merck | 1499230-935 |
| Methanol | Merck | 144282 |
| **Consumables** |  |  |
| Cell culture insert with a 0.4-μm pore | Corning, NY, USA | 3401 |
| PVDF membranes | Millipore, USA | [Cat. No. 88520](https://www.thermofisher.cn/order/catalog/product/88520) |
| Amicon Ultra-4 Centrifugal Filter Devices | Millipore, USA | Cat. No. UFC8010D |
| Waters ACQUITY UPLC BEH C18 1.7μm, 2.1 mm×100 mm column | Waters | SKU: 186002352 |
| **Mice** | Company | Strain |
| CD45.1 | Cyagen Biosciences | C57 strain |
| **Instrument Name** | Brand | Model |
| Becton Dickinson FACSCalibur Cytometer | BD Biosciences, USA | FACSCalibur |
| Leica TCS SP8 STED | German | TCS SP8 |
| Olympus phase‑contrast microscope | Japan | CKX53 |
| Transmission Electron Microscope (TEM) | FEI | TECNAI G212 |
| Biacore | Cytiva | 8K |
| Mass Spectrometer | AB SCIEX | 5500 QTRAP |
| Ultra-High Performance Liquid Chromatography (UHPLC) | Shimadzu | Nexera X2 LC-30AD |
| **Software** |  |  |
| GraphPad Prism |  |  |
| Analyst 1.6.3 software | SCIEX, Concord, Ontario, Canada | <https://sciex.com> |
| Biacore Insight Evaluation Software | Cytiva, Uppsala, Sweden |  |
| WinMDI vers 2.9 software |  |  |
| NIS Viewer software |  |  |
| Phenochart | Akoya Biosciences, Inc., Marlborough, MA, USA | v1.2.0 |
| inForm software | Akoya Biosciences, Inc., Marlborough, MA, USA | v2.6 |
| PhenoptrReports | Akoya Biosciences, Inc., Marlborough, MA, USA | v0.3.2 |

**Supplementary Table S3** Plasmids and Sequences

| siRNA | SENSE | ANTISENSE |
| --- | --- | --- |
| SMAD5 | CCTGGAAAGUUCUUUGGAAUU | AAUUCCAAAGAACUUUCCAGG |
| Control siRNA sequence | GUGAGCGUCUAUAUACCAUTT | AUGGUAUAUAGACGCUCACTT |
| mimic miR-21-3p | UCAACAUCAGUCUGAUAAGCUA |  |
| anti-miR-21-3p | AGCUUAUCAGACUGAUGUUGA |  |
| Negative Control | Random sequence (Provided by company) |  |
| WT full length (SacI-XbaI) | CCGGAGCTCTCATACTCAACTGGGGGAAGGGGTGCTCCTTAAATATATATAGATATGTATATATACATGTTTTTCTATTAAAAATAGACAGTAAAATACTATTCTCATTATGTTGATACTAGCATACTTAAAATATCTCTAAAATAGGTAAATGTATTTAATTCCATATTGATGAAGATGTTTATTGGTATATTTTCTTTTTCGTCTATATATACATATGTAACAGTCAAATATCATTTACTCTTCTTCATTAGCTTTGGGTGCCTTTGCCACAAGACCTAGCCTAATTTACCAAGGATGAATTCTTTCAATTCTTCATGCGTGCCCTTTTCATATACTTATTTTATTTTTTACCATAATCTTATAGCACTTGCATCGTTATTAAGCCCTTATTTGTTTTGTGTTTCATTGGTCTCTATCTCCTGAATCTAACACATTTCATAGCCTACATTTTAGTTTCTCTATCTAGA |  |
| MT full length (GATGTTT→GAGACCT) | CCGGAGCTCTCATACTCAACTGGGGGAAGGGGTGCTCCTTAAATATATATAGATATGTATATATACATGTTTTTCTATTAAAAATAGACAGTAAAATACTATTCTCATTATGTTGATACTAGCATACTTAAAATATCTCTAAAATAGGTAAATGTATTTAATTCCATATTGATGAAGAGACCTATTGGTATATTTTCTTTTTCGTCTATATATACATATGTAACAGTCAAATATCATTTACTCTTCTTCATTAGCTTTGGGTGCCTTTGCCACAAGACCTAGCCTAATTTACCAAGGATGAATTCTTTCAATTCTTCATGCGTGCCCTTTTCATATACTTATTTTATTTTTTACCATAATCTTATAGCACTTGCATCGTTATTAAGCCCTTATTTGTTTTGTGTTTCATTGGTCTCTATCTCCTGAATCTAACACATTTCATAGCCTACATTTTAGTTTCTCTATCTAGA |  |
